# Single-swap editing for the correction of common Duchenne muscular dystrophy mutations

**DOI:** 10.1016/j.omtn.2023.04.009

**Published:** 2023-04-19

**Authors:** Andreas C. Chai, Francesco Chemello, Hui Li, Takahiko Nishiyama, Kenian Chen, Yu Zhang, Efraín Sánchez-Ortiz, Adeeb Alomar, Lin Xu, Ning Liu, Rhonda Bassel-Duby, Eric N. Olson

**Affiliations:** 1 Department of Molecular Biology, University of Texas Southwestern Medical Center, Dallas, TX 75390, USA; 2 Hamon Center for Regenerative Science and Medicine, University of Texas Southwestern Medical Center, Dallas, TX 75390, USA; 3 Quantitative Biomedical Research Center, Department of Population and Data Sciences, University of Texas Southwestern Medical Center, Dallas, TX 75390, USA

**Keywords:** MT: RNA/DNA editing, base editing, CRISPR-Cas9, gene editing, exon skipping, DMD, Duchenne muscular dystrophy, AAV, iPSC, iPSC-CM

## Abstract

Duchenne muscular dystrophy (DMD) is a fatal X-linked recessive disease of progressive muscle weakness and wasting caused by the absence of dystrophin protein. Current gene therapy approaches using antisense oligonucleotides require lifelong dosing and have limited efficacy in restoring dystrophin production. A gene editing approach could permanently correct the genome and restore dystrophin protein expression. Here, we describe single-swap editing, in which an adenine base editor edits a single base pair at a splice donor site or splice acceptor site to enable exon skipping or reframing. In human induced pluripotent stem cell-derived cardiomyocytes, we demonstrate that single-swap editing can enable beneficial exon skipping or reframing for the three most therapeutically relevant exons—*DMD* exons 45, 51, and 53—which could be beneficial for 30% of all DMD patients. Furthermore, an adeno-associated virus delivery method for base editing components can efficiently restore dystrophin production locally and systemically in skeletal and cardiac muscles of a DMD mouse model containing a deletion of *Dmd* exon 44. Our studies demonstrate single-swap editing as a potential gene editing therapy for common DMD mutations.

## Graphical abstract

**Figure:**
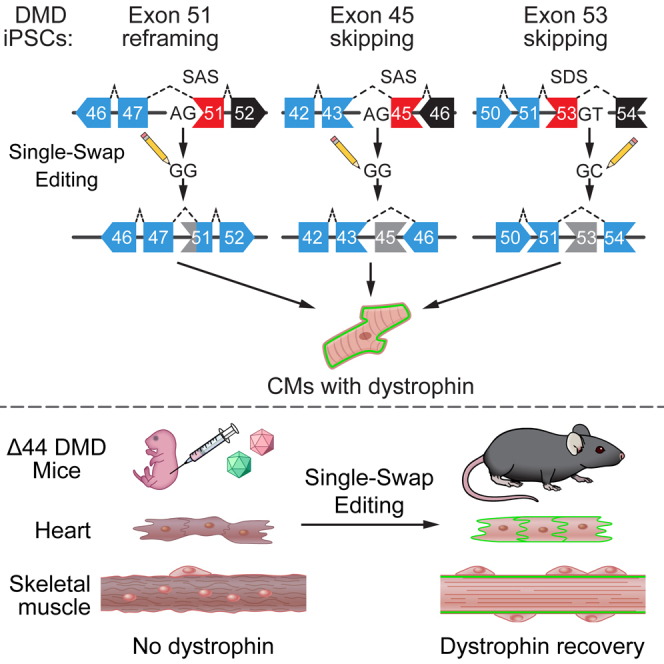

## Teaser

Olson and colleagues demonstrate that a single-swap base edit of splice acceptor or donor sites can cause skipping of the immediately adjacent exon. In cell and animal models of Duchenne muscular dystrophy (DMD), exon skipping can therapeutically restore dystrophin protein expression, highlighting an alternative therapeutic strategy.

## Introduction

Duchenne muscular dystrophy (DMD) is an X-linked recessive disease of progressive neuromuscular weakness and wasting that affects approximately 1 in 5,000 boys.^1^ While current advancements in clinical care have improved the survival of DMD patients, there is no cure for DMD, and death usually occurs due to cardiac or respiratory failure by the patient’s 20s–30s.^2^ New therapies and potential cures are urgently needed. DMD is caused by mutations in the *DMD* gene that result in absent functional dystrophin protein.^3^ Although *DMD* is the largest human gene, with thousands of documented clinical mutations, exon deletion mutations account for over 70% of all DMD cases.^4^ Furthermore, mutations tend to occur within two hotspots of the *DMD* gene: between exons 2 and 9 and exons 43 and 55. Exon deletions within these hotspots cause frameshift mutations and production of nonfunctional truncated dystrophin protein. Previous studies^5^^,^^6^^,^^7^ have deployed CRISPR-Cas9 nuclease gene editing strategies to induce a single double-stranded DNA break (DSB) that, when repaired by nonhomologous end joining (NHEJ), can introduce small insertions or deletions of DNA base pairs that can reframe the transcript or skip entire exons by disrupting splice acceptor sites. These reframing or exon skipping events can restore the production of truncated, but partially functional, dystrophin protein for a subset of DMD mutations.

Base editing has emerged as an attractive method to correct and potentially cure genetically based diseases. Base editors are fusion proteins of Cas9 nickase or deactivated Cas9 and an engineered deaminase protein, which allow base-pair edits within a defined editing window in relation to the protospacer adjacent motif (PAM) site of a single-guide RNA (sgRNA).^8^^,^^9^ Adenine base editors (ABEs) use deoxyadenosine deaminase to convert DNA A⋅T base pairs to G⋅C base pairs via an inosine intermediate. Cytosine base editors (CBEs) use cytidine deaminases to convert DNA C⋅G base pairs to T⋅A base pairs via a uracil intermediate. Our group and others have shown the potential of base editors to treat DMD by correcting point mutations in the *DMD* gene^10^^,^^11^ or by causing exon skipping via single-swap editing of splice sites.^12^^,^^13^ These base editing approaches may have advantages over CRISPR-Cas9 nuclease single-cut approaches by permanently correcting the genome without causing DNA DSBs, which have been shown to be deleterious to cells.^14^^,^^15^^,^^16^ Similarly, the ability of a base editor to permanently enable therapeutic exon skipping offers advantages over antisense oligonucleotide (ASO) approaches, which require lifelong dosing and are very inefficient.^17^

In single-swap editing, base editors induce single-base-pair changes at either the splice acceptor site (SAS) or the splice donor site (SDS) flanking a target exon.^12^ Both ABEs and CBEs can edit the canonical ^5′^AG^3′^ splice site of the SAS (^5’^CT^3’^ on the antisense strand) or the canonical ^5’^GT^3’^ splice site of the SDS (^5′^AC^3′^ on the antisense strand). Disruption of one of these splice sites by swapping out one of these bases for another base prevents the spliceosome from pairing the splice sites flanking an exon, thereby skipping a target exon in the final mature mRNA transcript.^18^

Here we report the use of an ABE-mediated gene editing strategy for single-swap editing as a correction strategy for the three most therapeutically relevant exons—*DMD* exons 45, 51, and 53—in human induced pluripotent stem cell (iPSC)-derived cardiomyocytes (iPSC-CMs), which could be beneficial for nearly 30% of all DMD patients.^19^ Single-swap editing restores dystrophin production in these human cell models of DMD. Furthermore, we demonstrate systemic delivery of adeno-associated virus (AAV)-mediated single-swap exon skipping components to correct both skeletal muscles and the heart of a mouse model of DMD and restore functional dystrophin protein production.

## Results

### Single-swap editing of *DMD* exon 51 induces beneficial exon reframing

**Figure 1:**
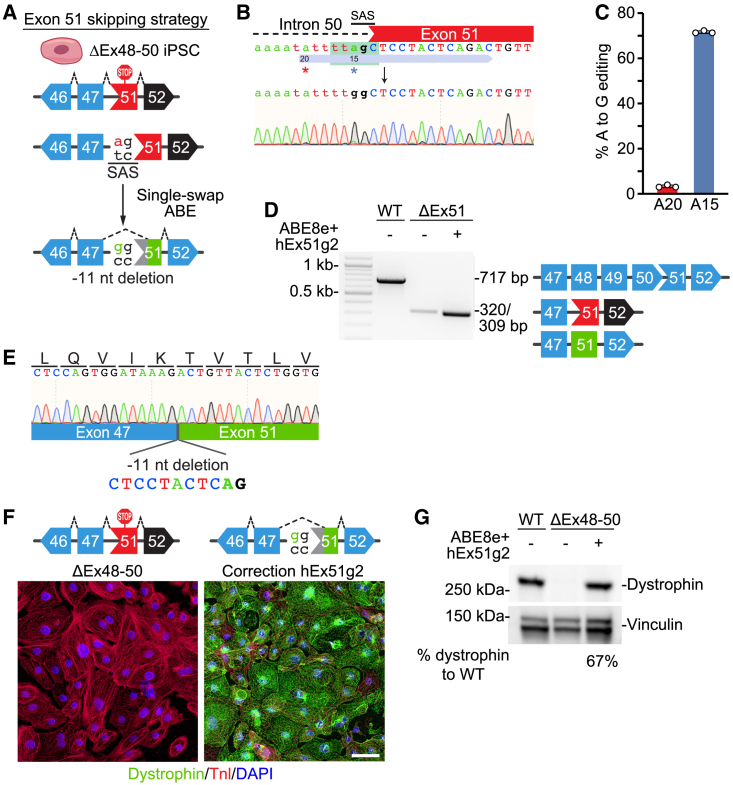
Single-swap editing at the SAS of *DMD* exon 51 induces beneficial exon reframing in ΔEx48–50 iPSC-CMs (A) Single-swap editing at the canonical ^5′^AG^3′^ SAS around human *DMD* exon 51 activates a cryptic splice acceptor site within exon 51 that causes an 11 nt deletion of the mature transcript. In ΔEx48–50 iPSC-CMs, this 11 nt deletion restores the open reading frame. (B) Schematic of hEx51g2 targeting the SAS of *DMD* exon 51 and representative chromatogram following editing using ABE8e-nSpCas9-NG. Editable adenines are indicated by asterisks: target adenine is at position 15; bystander adenine, position 20. Editing window is in green. (C) Editing efficiency by Sanger sequencing of hEx51g2 with ABE8e-nSpCas9-NG in ΔEx48–50 iPSCs at editable adenines for *DMD* exon 51 SAS. Target adenine is colored in blue; editing efficiency is 71.6% ± 0.6%. Bystander adenine is colored in red. n = 3 independent replicates. (D) RT-PCR analysis of mRNA from WT, ΔEx48–50, and ΔEx48–50 edited with ABE8e-nSpCas9-NG and hEx51g2 iPSC-CMs. The cDNA of the WT is 717 bp, of the ΔEx48–50 is 320 bp, and of the ΔEx48–50 with ABE8e-nSpCas9-NG and hEx51g2 is 309 bp. (E) Sanger sequencing of the cDNA from the ΔEx48–50 iPSC-CMs edited with ABE8e-nSpCas9-NG and hEx51g2 reveals splicing of *DMD* exon 47 to exon 51 with an 11 bp deletion. (F) Immunocytochemistry of ΔEx48–50 iPSC-CMs edited with ABE8e-nSpCas9-NG and hEx51g2 shows restoration of dystrophin protein. Dystrophin is in green; cardiac troponin I (TnI) highlights CMs in red; DAPI stains for nuclei in blue. Scale bar, 50 μm. (G) Western blot of ΔEx48–50 iPSC-CMs edited with ABE8e-nSpCas9 and hEx51g2 shows restoration of dystrophin protein. Vinculin is the loading control. Relative intensity is measured as dystrophin expression normalized to vinculin compared with the WT. Data are mean ± SD.

We first sought to develop a single-swap editing strategy to skip *DMD* exon 51, which could restore dystrophin in 13% of DMD patients.^19^ As ABEs have an optimal activity window in protospacer positions 13–17^20^ (counting the first nucleotide immediately 5′ of the PAM sequence as protospacer position 1), we designed three human sgRNAs with NG PAMs for exon 51 that place the SAS or SDS within the optimal activity window (Figure S1A). We opted to use the engineered deaminase ABE8e,^21^ a highly processive adenosine deaminase that has a wide editing window, fused to the engineered nSpCas9-NG variant nickase that recognizes NG PAMs^22^ (ABE8e-nSpCas9-NG). Following transient transfection via lipofection of HEK293T cells with ABE8e-nSpCas9-NG and each of the sgRNAs, we identified hEx51g2 as the most efficient sgRNA to induce base editing of the SAS of exon 51 (Figure S1B). As HEK293Ts do not highly express the *DMD* transcript or dystrophin protein, we next moved our system to human iPSCs, which can be differentiated into *DMD*-expressing CMs, to determine if single-swap editing of the SAS of exon 51 could induce exon skipping. We took patient-derived iPSCs containing a deletion of *DMD* exons 48–50 (ΔEx48–50), for which skipping of exon 51 could restore dystrophin protein production (Figure 1A), and nucleofected them with plasmids for ABE8e-nSpCas9-NG and hEx51g2. By Sanger sequencing, we found an editing efficiency of 71.6% ± 0.6% of the target A to G in the SAS of exon 51, with minimal bystander editing of 3.3% ± 0.6% of A20 (Figures 1B and 1C). Sanger sequencing of *in silico*-predicted candidate off-target sites revealed minimal to no off-target editing (<0.2%) (Figures S1C and S1D), suggesting that the hEx51g2 and ABE8e-nSpCas9-NG base editing system was highly efficient and specific for the SAS of exon 51. We took the pool of nucleofected iPSCs and differentiated them into CMs to determine if dystrophin expression was restored. By RT-PCR analysis, we did not detect the expected shift in band size caused by exon 51 skipping (Figure 1D). Sanger sequencing of the cDNA revealed an 11 nucleotide (nt) deletion at the beginning of exon 51, due to activation of a cryptic SAS downstream of the canonical SAS (Figure 1E). While single-swap editing of the SAS of *DMD* exon 51 did not result in exon skipping, activation of this cryptic splice site and the consequent 11 nt deletion in the mature mRNA resulted in beneficial exon reframing that restored dystrophin expression in differentiated CMs as demonstrated by immunocytochemistry (ICC) and western blot for dystrophin protein (Figures 1F and 1G).

### Single-swap editing of *DMD* exon 45 or exon 53 induces beneficial exon skipping

We next sought to perform single-swap exon skipping of *DMD* exons 45 and 53, which could each theoretically restore dystrophin protein production in 8% of DMD patients.^19^

#### Single-swap editing of exon 45

**Figure 2:**
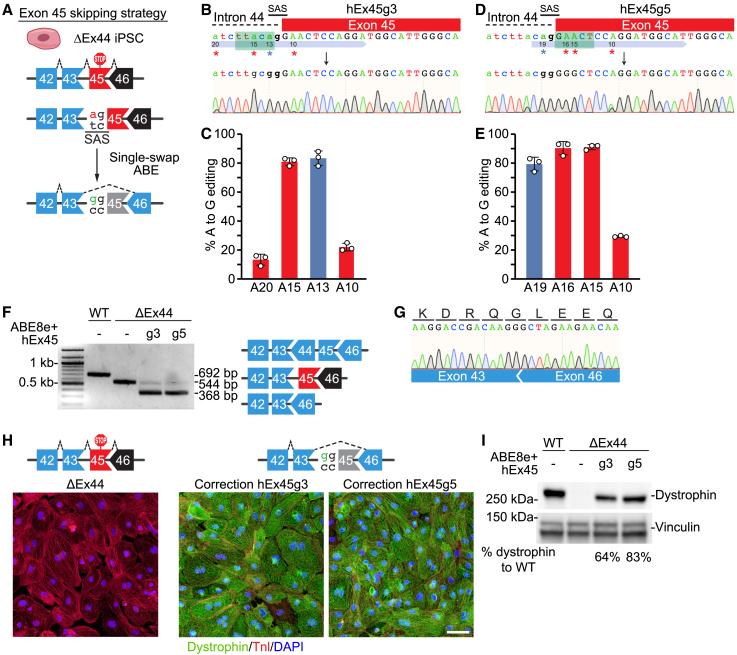
Single-swap editing at the SAS of *DMD* exon 45 induces beneficial exon skipping in ΔEx44 iPSC-CMs (A) Single-swap editing at the canonical ^5’^AG^3’^ SAS around human *DMD* exon 45 causes exon skipping. In ΔEx44 iPSC-CMs, skipping of exon 45 restores the open reading frame. (B) Schematic of hEx45g3 targeting the SAS of *DMD* exon 45 and representative chromatogram following editing using ABE8e-nSpCas9. Editable adenines are indicated by asterisks: target adenine is at position 13; bystander adenine, positions 10, 15, and 20. Editing window is in green. (C) Editing efficiency by Sanger sequencing of hEx45g3 with ABE8e-nSpCas9 in ΔEx44 iPSCs at editable adenines for *DMD* exon 45 SAS. Target adenine is colored in blue; editing efficiency is 83.3% ± 5.0%. Bystander adenines are colored in red. n = 3 independent replicates. (D) Schematic of hEx45g5 targeting the SAS of *DMD* exon 45 and representative chromatogram following editing using ABE8e-nSpCas9. Editable adenines are indicated by asterisks: target adenine is at position 19; bystander adenine, positions 10, 15, and 16. Editing window is in green. (E) Editing efficiency by Sanger sequencing of hEx45g5 with ABE8e-nSpCas9 in ΔEx44 iPSCs at editable adenines for *DMD* exon 45 SAS. Target adenine is colored in blue; editing efficiency is 79.3% ± 4.7%. Bystander adenines are colored in red. n = 3 independent replicates. (F) RT-PCR analysis of mRNA from WT, ΔEx44, and ΔEx44 edited with ABE8e-nSpCas9 and either hEx45g3 or hEx45g5 iPSC-CMS. The cDNA of the WT is 692 bp, of the ΔEx44 is 544 bp, and of the ΔEx44 with exon skipping of exon 45 is 368 bp. (G) Sanger sequencing of the 368 bp cDNA band shows splicing of *DMD* exon 43 to exon 46. (H) Immunocytochemistry of ΔEx44 iPSC-CMs edited with ABE8e-nSpCas9 and either hEx45g3 or hEx45g5 shows restoration of dystrophin protein. Dystrophin is in green; cardiac troponin I (TnI) highlights CMs in red; DAPI stains for nuclei in blue. Scale bar, 50 μm. (I) Western blot of ΔEx44 iPSC-CMs edited with ABE8e-nSpCas9 and either hEx45g3 or hEx45g5 shows restoration of dystrophin protein. Vinculin is the loading control. Relative intensity is measured as dystrophin expression normalized to vinculin compared with the WT. Data are mean ± SD.

We first designed six human sgRNAs with NG or NGG PAMs that target the SAS and two human sgRNAs with NG PAMs that target the SDS of *DMD* exon 45 (Figure S2A). By transient transfection via lipofection of candidate sgRNAs with either ABE8e-nSpCas9-NG or ABE8e-nSpCas9, which recognizes NGG PAMs, we identified hEx45g3 and hEx45g5 as the best candidate sgRNAs due to their high efficiencies in base editing the SAS of exon 45 (Figure S2B). We then took patient-derived iPSCs containing deletion of *DMD* exon 44 (ΔEx44), for which skipping of exon 45 could restore dystrophin protein expression (Figure 2A), and nucleofected them with hEx45g3 or hEx45g5 and ABE8e-nSpCas9, as the sgRNAs hEx45g3 and hEx45g5 have NGG PAMs. By Sanger sequencing, we found that hEx45g3 and hEx45g5 with ABE8e-nSpCas9 had similar editing efficiencies of the target A to G of the exon 45 SAS (83.3% ± 5.0% for A13 and 79.3% ± 4.7% for A19, respectively). Both hEx45g3 and hEx45g5 had significant bystander editing of adenines, especially of those within the canonical editing windows (from 13.3% ± 3.8% to 81.0% ± 2.6%, and 29.3% ± 0.6% to 91.0% ± 1.7%, respectively) (Figures 2B–2E). However, as these edits occur within the intron or to-be-skipped exon, these bystander edits do not carry over into the final mature transcript. We then looked at potential DNA off-target editing and found that hEx45g3 had significant off-target activity at two of the top five predicted sites (7%–8%) by Sanger sequencing (Figures S2C and S2D). Both sites occur in the intronic region, and potential consequences remain to be determined. We found that for hEx45g5, there were no significant editing events in the top five predicted off-target sites (Figures S2E and S2F) by Sanger sequencing. We took both populations of nucleofected iPSCs and differentiated them into CMs. For both hEx45g3 and hEx45g5, RT-PCR analysis showed the expected shift in band size as a result of exon 45 skipping (Figure 2F), and Sanger sequencing confirmed the skipping of exon 45 in the ΔEx44 iPSCs as exon 43 spliced into exon 46 (Figure 2G). Skipping of exon 45 in these ΔEx44 iPSC-CMs restored dystrophin protein expression as demonstrated by ICC and western blot (Figures 2H and 2I).

#### Single-swap editing of exon 53

**Figure 3:**
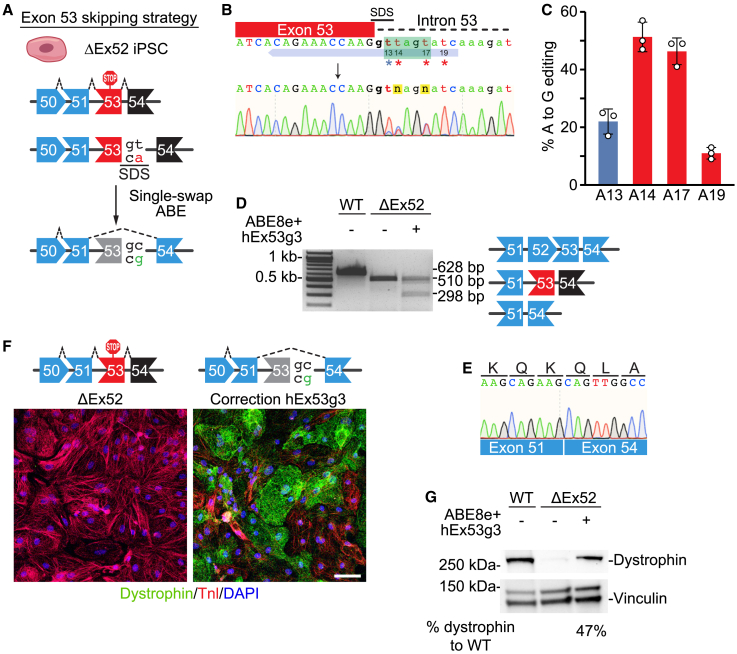
Single-swap editing at the SDS of *DMD* exon 53 induces beneficial exon skipping in ΔEx52 iPSC-CMs (A) Single-swap editing on the antisense strand of the canonical ^5′^GT^3′^ SDS around human *DMD* exon 53 causes exon skipping. In ΔEx52 iPSC-CMs, skipping of exon 53 restores the open reading frame. (B) Schematic of hEx53g3 targeting the SDS of *DMD* exon 53 and representative chromatogram following editing using ABE8e-nSpCas9-NG. Editable adenines are indicated by asterisks: the target adenine is at position 13; bystander adenine, positions 14, 17, and 19. Editing window is in green. (C) Editing efficiency by Sanger sequencing of hEx53g3 with ABE8e-nSpCas9-NG in ΔEx52 iPSCs at editable adenines for *DMD* exon 53 SDS. Target adenine is colored in blue; editing efficiency is 22.0% ± 4.4%. Bystander adenines are colored in red. n = 3 independent replicates. (D) RT-PCR analysis of mRNA from WT, ΔEx52, and ΔEx52 edited with ABE8e-nSpCas9-NG and hEx52g3 iPSC-CMs. The cDNA of the WT is 628 bp, of the ΔEx52 is 510 bp, and of the ΔEx52 with exon skipping of exon 53 is 298 bp. (E) Sanger sequencing of the 298 bp cDNA band shows splicing of *DMD* exon 51 to exon 54. (F) Immunocytochemistry of ΔEx52 iPSC-CMs edited with ABE8e-nSpCas9-NG and hEx53g3 shows restoration of dystrophin protein. Dystrophin is in green; cardiac troponin I (TnI) highlights CMs in red; DAPI stains for nuclei in blue. Scale bar, 50 μm. (G) Western blot of ΔEx52 iPSC-CMs edited with ABE8e-nSpCas9 and hEx53g3 shows restoration of dystrophin protein. Vinculin is the loading control. Relative intensity is measured as dystrophin expression normalized to vinculin compared with the WT. Data are mean ± SD.

For skipping of *DMD* exon 53, we designed two human sgRNAs with NG PAMs targeting the SAS of exon 53 and two human sgRNAs with NG PAMs targeting the SDS of exon 53 (Figures 3A and S3A). Transient transfection via lipofection of HEK293T cells with candidate sgRNAs and ABE8e-NG suggested that hEx53g3 targeting the SDS was the most efficient in disrupting one of the splice sites around exon 53 (Figure S3B). We then took patient-derived iPSCs lacking *DMD* exon 52 (ΔEx52) and nucleofected them with plasmids for hEx53g3 and ABE8e-NG. By Sanger sequencing, editing efficiency of the target A to G was 22.0% ± 4.4% with hEx53g3, which was lower than the best sgRNAs for exon 45 and 51, potentially due to innate nucleotide sequence differences in the sgRNAs^23^ or use of the less efficient nSpCas9-NG variant^24^ (Figures 3B and 3C). Sanger sequencing revealed no significant editing events in the top five predicted off-target sites (Figures S3C and S3D). We then differentiated the nucleofected ΔEx52 iPSCs to CMs to analyze dystrophin protein expression. By RT-PCR analysis we observed a shift in band size as a result of exon skipping (Figure 3D), and Sanger sequencing analysis confirmed skipping of exon 53 in the *DMD* ΔEx52 iPSC-CMs as *DMD* exon 51 was spliced into exon 54 (Figure 3E). In these edited *DMD* ΔEx52 iPSC-CMs, exon 53 skipping also restored dystrophin expression as demonstrated by ICC and western blot (Figures 3F and 3G) to levels expected of the editing efficiency.

### Design of an effective delivery method for *in vivo* single-swap editing in the DMD ΔEx44 mouse model

Having demonstrated that single-swap editing can correct various exon deletion mutations by skipping three of the most therapeutically relevant exons, we next sought to test our single-swap system *in vivo*. We used mice containing a deletion of exon 44 (ΔEx44 mice)^5^ to conduct exon skipping of exon 45 by single-swap editing. As the SAS region of *DMD* exon 45 is highly homologous between human and mouse (Figure S4A), we could use the same highly efficient sgRNAs that we identified in our initial *in vitro* screen for the human genome. Accordingly, hEx45g5 could be used in the mouse locus without any modification (hmEx45g5), whereas we shortened hEx45g3 to 18 nt (canonical length is 20 nt) to now be homologous between the mouse and the human sequences (hmEx45g3-18nt).

To optimize our single-swap editing strategy for *in vivo* testing, we screened three different highly processive adenosine deaminases fused to nSpCas9 for their efficiencies with hmEx45g5 and hmEx45g3-18nt: ABE8e, a phage-assisted evolved variant of the foundational ABE7.10 variant^21^; ABE8eV106W,^21^ an ABE8e variant with reduced RNA and DNA off-target editing; and ABE8.20m,^25^ an engineered variant of the foundational ABE7.10 variant, independently discovered from ABE8e. In mouse C2C12 myoblasts, by transient transfection via lipofection, we found that hmEx45g3-18nt with ABE8e or ABE8eV106W was the most efficient in editing the target adenine (49.5% ± 1.7%, and 47.7% ± 3.1%, respectively) by Sanger sequencing (Figure S4B). As the editing efficiency was similar for hmEx45g3-18nt with ABE8e and ABE8eV106W, we opted to use the ABE8eV106W variant to reduce potential RNA and Cas-independent DNA off-target editing.

**Figure 4:**
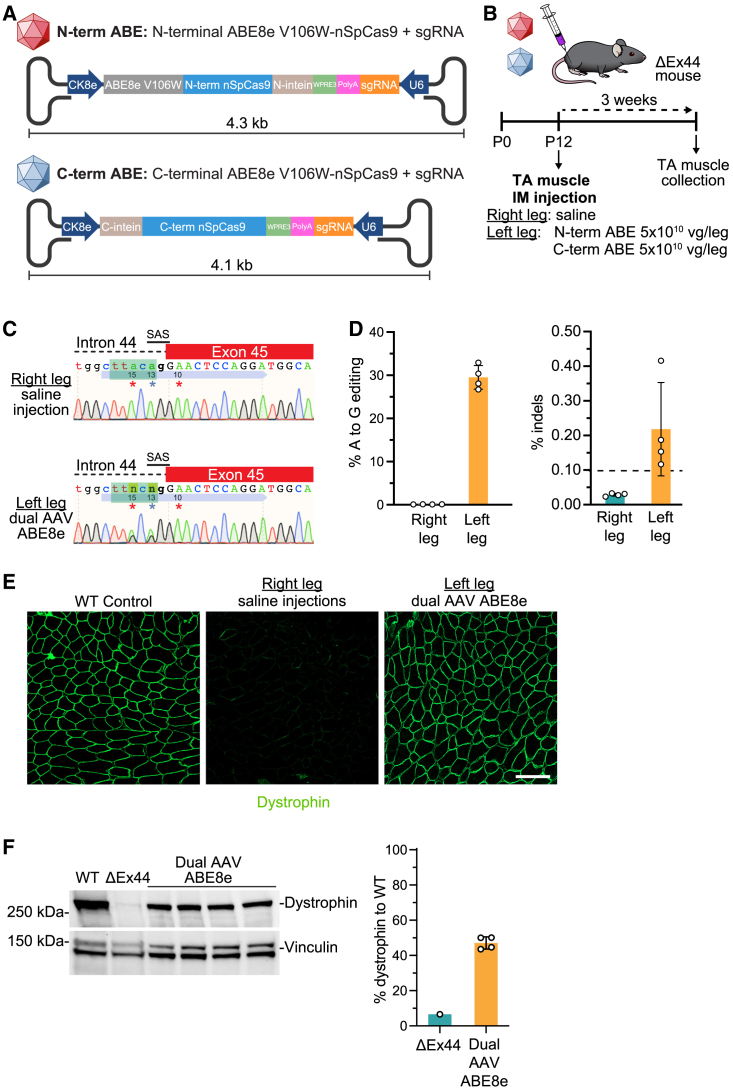
Intramuscular injection of a single-swap editing dual AAV system restores dystrophin protein production in a ΔEx44 mouse model of DMD (A) Schematic for the dual AAV ABE8e system. The CK8e muscle-specific promoter drives expression of ABE8eV106W-nSpCas9 base editor halves and their intein tags for protein *trans*-splicing. Each viral construct also contains the woodchuck hepatitis post-transcriptional regulatory element (WPRE3), a synthetic mini polyadenylation signal (PolyA), and an hU6 promoter-sgRNA cassette (U6-sgRNA). (B) At P12, ΔEx44 mice received saline in the right leg and the dual AAV ABE8e system in the left leg by intramuscular injection into the TA muscle. Three weeks post-injection, TA muscles were collected. (C) Representative Sanger sequencing chromatograms of genomic DNA from the right leg 3 weeks post-saline injection (top) and from the left leg 3 weeks post-dual AAV ABE8e treatment (bottom). Blue arrow indicates the hmEx45g3-18nt sgRNA. Target adenine is at position 13. Bystander adenines are at positions 10 and 15. Editing window is in green. (D) Editing efficiency of target adenine and indel frequency by amplicon deep sequencing in genomic DNA from the left TA 3 weeks post-dual AAV ABE8e treatment. Target adenine is A13 (blue); editing efficiency is 29.5% ± 2.7% in the left leg. Indel frequency is 0.2% ± 0.1% in the left leg. n = 4 mice. (E) Immunohistochemistry for dystrophin expression from the saline-injected right leg and the dual AAV ABE8e-injected left leg from a ΔEx44 mouse and the TA of a WT mouse. Scale bar, 100 μm. Dystrophin is stained in green. (F) Western blot and (G) quantification of dystrophin protein expression from the left leg of a WT mouse, a ΔEx44 mouse, and four dual AAV ABE8e-injected ΔEx44 mice (47.1% ± 3.5%). Vinculin is the loading control. Relative intensity is measured as dystrophin expression normalized to vinculin compared with the WT. n = 1–4 mice. Data are mean ± SD.

For *in vivo* delivery, we packaged expression units encoding ABE8eV106W-nSpCas9 and hmEx45g3-18nt within AAV capsids, a commonly used viral delivery method. We chose to use the AAV9 capsid for its high cardiac and skeletal muscle transduction ability and use in clinical trials.^26^ We used the muscle-specific CK8e promoter^27^ to further limit expression of the base editor to cardiac and skeletal muscle. However, inclusion of the full-length base editor (∼4.8 kb), the CK8e promoter (∼0.4 kb), synthetic mini poly(A) (0.06 kb), U6 promoter (0.24 kb), and sgRNA (0.11 kb)—the minimum components needed for base editing—exceeds the packaging limit of a single AAV9 (∼4.7 kb). Consequently, we split the base editor coding sequences across two AAV9s and used *trans-*splicing inteins^28^ to reconstitute the full-length ABE8eV106W-nSpCas9 in cells upon protein expression, and we confirmed this assembly in transfected HEK293T cells (Figure S4C). For this dual AAV system (dual AAV ABE8e), each AAV half also contained a single copy of a sgRNA expression cassette for hEx45g3-18nt (Figure 4A). We validated our dual AAV ABE8e in C2C12 myotubes and found that the base editor localized to the nucleus of the myotubes as expected (Figure S4D). Furthermore, we could achieve an editing efficiency of approximately 22.7% ± 1.5% of the target adenine by Sanger sequencing, suggesting that our dual AAV ABE8e was functional (Figure S4E).

To further validate our dual AAV ABE8e *in vivo*, we performed intramuscular injection in the tibialis anterior (TA) muscle of ΔEx44 mice. The left TA of post-natal day 12 (P12) ΔEx44 mice was injected with dual AAV ABE8e (5 × 10^10^ vg/leg of each viral half, 1 × 10^11^ vg total), while the right leg was injected with saline as a control. Tissues were collected 3 weeks after injection (Figure 4B). By amplicon deep sequencing (ADS) of the on-target site, we did not detect any editing (0.1% ± 0.0%) in the SAS of exon 45 in DNA extracted from the saline-injected control right leg, while we observed 29.5% ± 2.7% A to G editing of the target A in DNA extracted from the dual AAV ABE8e-injected left leg (Figures 4C and 4D). Insertions and deletions (indels) are a potential by-product of base editors, and we detected minimal indels at the SAS (0.2% ± 0.1%). At the top five predicted off-target sites (OTSs) (Figure S5A), we detected minimal editing (<0.1%, the limit of detection for next-generation sequencing (NGS)) at almost all candidate adenine sites. However, we detected editing by ADS at A15 of OTS2 (0.3% ± 0.1%) and A13 and A15 of OTS4 (0.2% ± 0.2% and 0.2% ± 0.2%, respectively) (Figure S5B). Importantly, we did not detect any viral genome integration at the on-target site (Figure S5C). Immunohistochemistry of transverse sections of the right TA showed complete absence of dystrophin, whereas almost all myofibers of the left TA were positive for dystrophin (Figures 4E and S6A). Furthermore, in the left TA, dystrophin was restored to almost 50% compared with wild type (WT) by western blot (Figure 4F). Skeletal muscle structure was also preserved, as we detected fewer markers of dystrophic muscle, including the absence of fibrosis and inflammation and a reduction of myofibers with centralized nuclei (Figures S6B and S6C).

### Single-swap editing systemically restores dystrophin expression in skeletal and cardiac muscle

**Figure 5:**
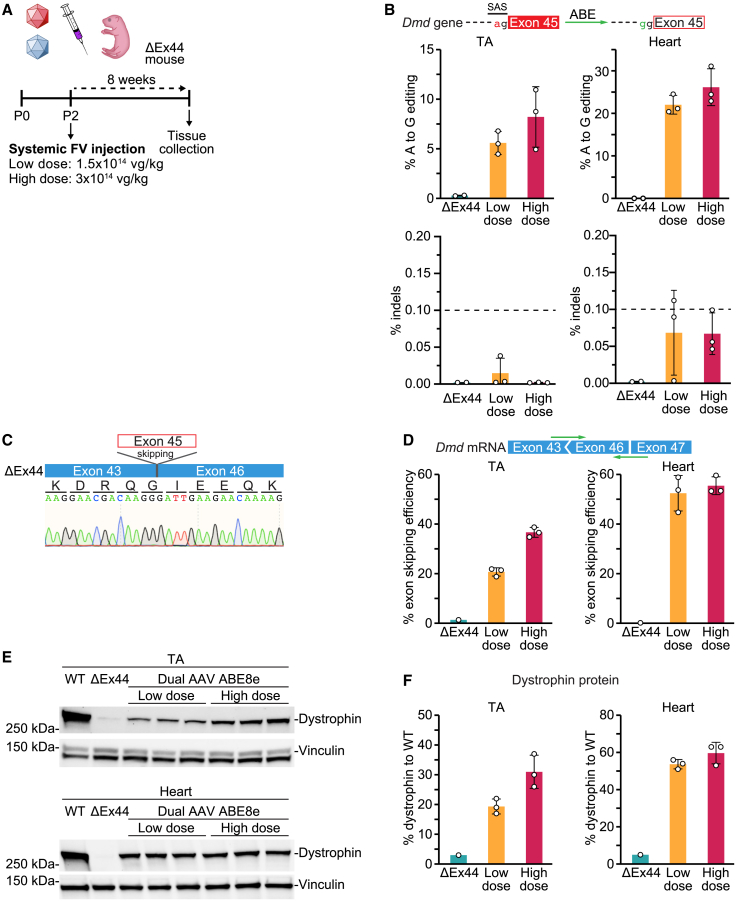
Systemic injection of a single-swap editing dual AAV system in a ΔEx44 mouse model of DMD restores dystrophin protein production in skeletal and cardiac muscles (A) At P2, ΔEx44 mice received systemically either a low dose or a high dose of the dual AAV ABE8e system via injection into the temporal facial vein. Tissues were collected 8 weeks later. (B) Editing efficiency for target adenine and indel frequency by amplicon sequencing in genomic DNA from the TA (left, ΔEx44, 0.2% ± 0.0%; low dose, 5.5% ± 1.2%; high dose, 8.1% ± 3.0%) and heart (right, ΔEx44, 0.2% ± 0.0%; low dose, 22.0% ± 2.2%; high dose, 26.2% ± 4.4%) of ΔEx44 mice following saline or dual AAV ABE8e treatment at the two doses. n = 2–3 mice. (C) Sanger sequencing of cDNA of heart tissue from a dual AAV ABE8e-treated ΔEx44 mouse showing splicing of *Dmd* exon 43 to exon 46. (D) Efficiency of exon skipping in mature mRNA from the TA (left, ΔEx44, 1.4%; low dose, 20.7% ± 1.7%; high dose, 36.7% ± 2.0%) and heart (right, ΔEx44, 0.2%; low dose, 52.4% ± 7.2%; high dose, 55.5% ± 3.6%) following dual AAV ABE8e treatment at the two doses. n = 1–3 mice. (E) Western blot and (F) quantification of dystrophin protein expression from the TA (left in F, ΔEx44, 3.0%; low dose, 19.3% ± 2.5%; high dose, 31.0% ± 5.6%) and heart (right in F, ΔEx44, 2.0%; low dose, 36.0% ± 1.0%; high dose, 65.0% ± 14.4%) of a WT mouse, a ΔEx44 mouse, and three dual AAV ABE8e-injected ΔEx44 mice each at the low and high dose. Vinculin is the loading control. Relative intensity is measured as dystrophin expression normalized to vinculin compared with the WT. Data are mean ± SD. n = 1–3 mice.

As DMD is a systemic disease affecting both skeletal and cardiac muscle, we next sought to deploy our dual AAV ABE8e editing strategy to systemically restore dystrophin protein expression in cardiac and skeletal muscle. We injected ΔEx44 mice at P2 via the temporal facial vein with a low dose of virus (1.5 × 10^14^ vg/kg total) or a high dose of virus (3 × 10^14^ vg/kg total) and collected tissues 8 weeks later (Figure 5A). We conducted analyses on whole-heart tissue and TA tissue, as an example of skeletal muscle. By ADS of genomic DNA of the TA, we detected 5.5% ± 1.2% editing efficiency of the target adenine of the SAS of *Dmd* exon 45 at the low dose, and 8.1% ± 3.0% at the high dose, while in genomic DNA of the heart, we detected 22.0% ± 2.2% and 26.2% ± 4.4% editing for the low and high dose, respectively. Minimal indel frequency was detected in both heart and TA for both doses (<0.1%) (Figure 5B). To determine specificity of the CK8e promoter, we measured A to G editing of the target adenine of the SAS of *Dmd* exon 45 in the lung, liver, gonads, and spleen from mice treated with the high dose of dual AAV ABE8e (Figure S7A). Notable editing was detected in the liver (11.1% ± 5.9%), likely due to the high viral copy number (Figure S7B), with lower levels in other measured nonmuscle tissues (<0.2%). We found undetectable levels of AAV integration (<0.1%) at the on-target site (Figure S7C).

As our DNA editing efficiencies in the heart and TA muscle are diluted by resident nonmuscle cells,^29^ such as endothelial cells and fibro-adipogenic progenitors, which should not be edited by our muscle-specific CK8e promoter, we next sought to evaluate the level of correction in *Dmd* mRNA, which is predominantly expressed by muscle cells. To check the efficiency of exon skipping following single-swap SAS editing, we performed quantitative polymerase chain reaction (qRT-PCR) using a forward primer that specifically recognizes the new junction between *Dmd* exon 43 and exon 46, which would indicate effective skipping of exon 45 in the ΔEx44 mouse model (Figure 5C). In the TA, we detected 20.7% ± 1.7% of dystrophin transcripts with exon skipping after treatment with the low dose of dual AAV ABE8e and more than 36.7% ± 2.0% after treatment with the high dose. In the heart, we detected 52.4% ± 7.2% and 55.5% ± 3.6% exon skipping efficiency at the low and high dose, respectively (Figure 5D). We also detected the presence of an alternative splicing event, likely due to activation of an upstream cryptic SAS following disruption of the canonical SAS of exon 45 (Figures S7D and S7E). Usage of this cryptic splice site results in a 92 bp intronic inclusion that introduces a premature termination codon, resulting in no dystrophin protein production for this alternative transcript. Importantly, this may be a mouse-specific alternative splicing event, as we did not detect this alternative transcript within human CMs. We next quantified dystrophin protein recovery by western blot (Figure 5E). In the TA, dystrophin protein recovery was 19.3% ± 2.5% of the WT level for the low viral dose and about 31.0% ± 5.6% of the WT level for the high viral dose. Recovery of dystrophin protein in the heart was higher, at 53.7% ± 2.5% of WT for the low viral dose and 59.7% ± 5.7% of WT for the high viral dose (Figure 5F).

### Rescue of muscle structure and function after single-swap editing

**Figure 6:**
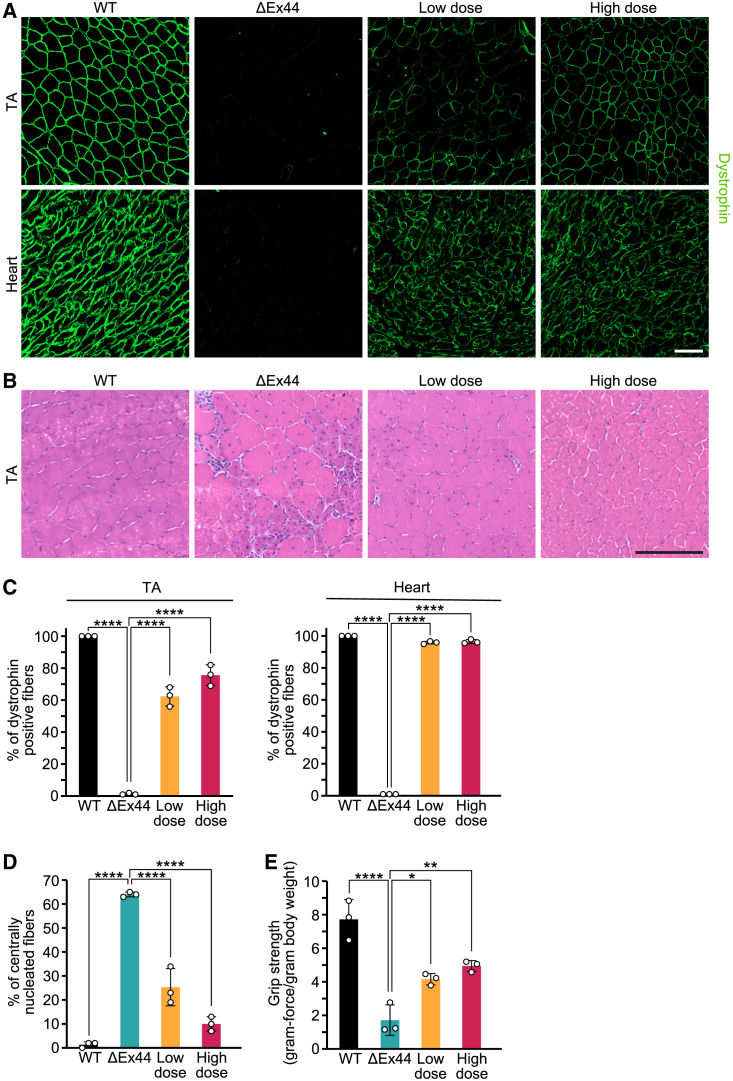
Single-swap editing restores functional dystrophin protein that rescues muscular dystrophy and weakness in a ΔEx44 mouse model of DMD (A) Immunohistochemistry for dystrophin from the TA and heart and (B) H&E staining from the TA of a WT mouse, a ΔEx44 mouse, and a dual AAV ABE8e-injected ΔEx44 mouse at the low and high dose. Scale bar, 100 μm. Dystrophin is stained in green. From WT mice, ΔEx44 mice, and dual AAV ABE8e-injected ΔEx44 mice at the low and high dose, quantification of (C) dystrophin-positive fibers in TA muscles (WT, 100.0% ± 0.0%; ΔEx44, 1.3% ± 0.6%; low dose, 62.3% ± 6.0%; high dose, 75.7% ± 6.5%) and heart (WT, 100.0% ± 0.0%; ΔEx44, 0.1% ± 0.0%; low dose, 95.2% ± 0.2%; high dose, 96.1% ± 0.4%), (D) centrally nucleated fibers in TA muscles (WT, 1.3% ± 1.2%; ΔEx44, 64.0% ± 1.0%; low dose, 25.3% ± 7.8%; high dose, 10.0% ± 3.0%), and (E) grip strength (WT, 7.7 ± 1.2 grams-force/grams body weight [gf/g]; ΔEx44, 1.7 ± 0.9 gf/g; low dose, 4.2 ± 0.3 gf/g; high dose, 4.9 ± 0.3 gf/g). Data are mean ± SD. n = 3 mice. ∗p < 0.05, ∗∗p < 0.01, ∗∗∗*∗*p < 0.0001 by ordinary one-way ANOVA.

We next sought to determine if recovery of partially truncated dystrophin protein (missing only *Dmd* exons 44 and 45) could prevent pathological muscle remodeling and restore muscle structure and function. Muscle sections from the TA and heart were stained by dystrophin immunohistochemistry or hematoxylin and eosin (H&E) (Figures 6A, 6B, S8A, and S8B). Approximately 62.3% ± 6.0% of muscle fibers were positive for dystrophin in the TA from mice injected with the low dose of virus, while more than 75.7% ± 6.5% of fibers were positive in mice injected with the high dose. In the heart, more than 95% of CMs were positive for dystrophin at both doses (Figure 6C). Furthermore, we observed an overall recovery of TA muscle structure. One of the hallmarks of dystrophic muscle is centralized nuclei within muscle fibers. In the TA of noninjected ΔEx44 mice, 64.0% ± 1.0% of fibers contained centralized nuclei. However, in ΔEx44 mice injected with the low dose of virus, only about 25.3% ± 7.8% of fibers contained centralized nuclei, and ΔEx44 mice injected with the high dose had 10.0% ± 3.0% of fibers with centralized nuclei (Figure 6D). Another hallmark of dystrophic muscle is an abnormal proportion of small myofibers, which are caused by repeated cycles of regeneration and degeneration and subsequent fibrosis and necrosis, and large myofibers, which are caused by compensatory hypertrophy.^30^ In TA muscles injected with both low and high doses, we saw an improvement in the distribution of fiber diameters. The standard deviation of fiber diameters was increased from 12.8 ± 8.0 μm in the WT mice to 17.5 ± 8.0 μm in the noninjected ΔEx44 mice. Treatment of ΔEx44 mice with the low dose reduced the standard deviation of fiber diameters to 10.8 ± 5.7 μm, and treatment with the high dose reduced the standard deviation to 11.9 ± 3.0 μm (Figure S8C). The percentage of fibrosis and necrosis was reduced from 14.0% ± 1.4% for the TA of noninjected ΔEx44 mice to about 7.0% ± 1.2% for the TA of ΔEx44 mice injected with the low dose and 5.9% ± 1.8% for the TA with the high dose (Figure S8D).

To determine whether single-swap editing exon skipping could restore muscle function, we performed grip strength analyses. Noninjected ΔEx44 mice showed a reduction in grip strength (1.7 ± 0.9 gram-force/gram body weight [gf/g]) compared with the WT (7.7 ± 1.2 gf/g) (22% of the WT strength). However, we detected an increase in strength of 31% (4.2 ± 0.3 gf/g) and 41% (4.9 ± 0.3 gf/g) for ΔEx44 mice injected with the low and high dose, respectively, compared with noninjected ΔEx44 mice (53% and 63% of the WT strength, respectively) (Figure 6E).

To extend our work further, we systemically treated juvenile P21 ΔEx44 mice with 3 × 10^14^ vg/kg total of our dual AAV ABE8e system via tail-vein injection (Figure S9A). By ADS, we could achieve similar or higher editing efficiencies of 10.3% ± 2.5% in the TA and 29.0% ± 1.7% in the heart of the target SAS adenine, with undetectable indel products and AAV integration events (<0.1%, the limit of detection for NGS) at the on-target site (Figure S9B). Treatment with the dual AAV ABE8e system restored dystrophin protein expression to 28.2% ± 9.8% of healthy control levels in the TA and 37.8% ± 4.3% of healthy control levels in the heart (Figure S9C). In the TA of treated ΔEx44 mice, 57.3% ± 3.8% of fibers expressed dystrophin, and there was a beneficial decrease in centrally nucleated fibers from 75.2% ± 2.1% in untreated mice to 45.1% ± 2.4% in treated mice (Figures S9D–S9G).

Taken together, these results suggest that single-swap editing can cause exon skipping and efficiently restore production of an internally truncated, but partially functional, dystrophin protein that ameliorates pathological features of muscular dystrophy.

## Discussion

In this preclinical study, we demonstrate the use of an ABE to induce single-swap editing at splice sites of *DMD* exons, thereby enabling exon skipping or reframing of the three most therapeutically relevant exons: *DMD* exons 45, 51, and 53. In human iPSC-CMs, single-swap disruption of the exon 51 SAS was not sufficient to induce its skipping, but caused the activation of a downstream cryptic SAS within exon 51 and an 11 nt partial exon exclusion of exon 51 in the final mature transcript. This deletion due to alternative splicing fortuitously reframed the dystrophin transcript and restored dystrophin protein expression in ΔEx48–50 iPSC-CMs. Future studies will seek to determine if this cryptic SAS is also activated in skeletal muscle and in cells derived from other patients. Exon skipping was achieved by efficient single-swap editing of both the SAS of exon 45 and the SDS of exon 53, which restored dystrophin protein in ΔEx44 and ΔEx52 iPSC-CMs, respectively. While moderate bystander editing is a characteristic drawback of base editing for the correction of point mutations, this drawback is negated in exon skipping applications, as the bystander edits occur in the intron or to-be-skipped exon and not in the mature transcript. Minimal editing was detected at two potential OTSs, likely due to the enhanced processive editing of the engineered ABE8e deaminase.^21^

Packaging of ABE8e-V106W-nSpCas9 and a truncated sgRNA, which can target both the human and the mouse genome, could efficiently single-swap edit the SAS of *Dmd* exon 45 in the ΔEx44 mouse model. Dystrophin restoration by exon skipping was detected at levels >30% of WT levels in TA muscle and >60% of WT levels in cardiac muscle at the highest dose per body weight currently used in US Food and Drug Administration (FDA)-approved clinical trials (NCT03199469). Dose-dependent production of this partially truncated dystrophin protein could prevent pathological remodeling of skeletal muscle fibers and restore grip strength in these mice. Treatment of juvenile ΔEx44 mice, which would better mirror ongoing clinical trials treating DMD patients in middle childhood (6–11 years), could also restore dystrophin levels to >25% of WT levels in TA muscle and >35% of WT levels in cardiac muscle and decrease pathological features, such as the percentage of centrally nucleated fibers. However, in DMD mice, the level of acute muscle necrosis and regeneration peaks at P21,^31^ causing potentially correctable myofibers to be replaced with noncorrectable fibrotic tissue. This limits the potential dystrophin recovery, as there are fewer and fewer muscle fibers available for correction, suggesting that earlier treatment may be more beneficial in DMD mouse models. We found significant editing in the liver tissue of dual AAV ABE8e-treated mice, but this was accompanied by nearly 20-fold higher viral copy numbers compared with the similarly edited TA muscle. These high viral copy numbers may be due to AAV9’s high transduction efficiency of the liver and increased vascular availability of the tissue. This suggests that the CK8e promoter may still be muscle specific with slight leakiness of activity in the liver tissue, but high viral copy numbers may lead to unintended high expression of the base editors and significant editing in nonmuscle tissues. Potential future clinical applications of this work may require the use of less leaky muscle-specific promoters or the use of liver-detargeting myotropic AAVs^32^^,^^33^ to minimize tissue expression in nonmuscle tissues. While our work represents a first step toward a cure for patients, future studies will need to investigate editing efficiencies in larger animal models such as nonhuman primates, optimizations in dose and delivery, and potential toxicology studies following AAV-mediated delivery of base editors.

Current FDA-approved clinical approaches for exon skipping rely on ASOs; there are four ASO treatments available for DMD patients that can induce skipping of *DMD* exon 45, 51, or 53. While these treatments remain the best available for the DMD patients of today, these ASO treatments require lifetime weekly intravenous infusions,^34^^,^^35^^,^^36^^,^^37^ restore low levels of dystrophin protein (<6%), and were initially approved based on the findings of low levels of dystrophin restoration rather than functional benefit.^17^ Our study suggests that ABE-mediated single-swap exon skipping could serve as a one-time therapy to restore high levels of functional dystrophin protein by targeting the same exons as current ASOs.

Previous proof-of-concept studies^5^^,^^6^^,^^7^^,^^38^^,^^39^^,^^40^^,^^41^^,^^42^ have demonstrated the use of CRISPR-Cas9 nuclease strategies to introduce single-cut DSBs at target *DMD* exons, which are then repaired by NHEJ, which can introduce indels and cause exon reframing or exon skipping and lead to beneficial restoration of functional dystrophin protein. As the NHEJ repair process is inherently error prone, only a fraction of repairs yields the correct reading frame, which may or may not lead to productive edits that reframe or skip the target *DMD* exon. While some indel outcomes may be more predominant over others,^43^ the indel outcomes are inherently heterogeneous and unpredictable. Furthermore, DSBs can lead to more frequent AAV integration events.^44^^,^^45^ Adenine base editing overcomes some of these limitations by having more defined editing outcomes (A to G base pair transitions within a defined 5 nt editing window) and no DSB formation, which minimizes potential AAV integration and indel formations. Our study adds to the growing body of work suggesting that base editing and other gene editing strategies could serve as potential future treatments for DMD.

## Materials and methods

### Study approval

All mouse experiments complied with all relevant ethical regulations and were performed according to protocols approved by the institutional animal care and use committees at the University of Texas Southwestern Medical Center (protocols 2016-101833 and 2017-102269). UT Southwestern uses the “Guide for the Care and Use of Laboratory Animals” when establishing animal research standards. All mice used in this study were housed at the pathogen-free Animal Resource Center at the University of Texas Southwestern Medical Center. All animals were bred inside a specific-pathogen-free facility with 12 h light:dark cycles with a temperature of 18°C–24°C and humidity of 35%–60% and monitored daily with no health problems. All animals were housed in groups of a maximum of five per cage with *ad libitum* access to food and water.

### Plasmids and vector construction

The plasmids for ABE8e (Addgene plasmid 138489),^21^ NG-ABE8e (Addgene plasmid 138491),^21^ and ABE8e(TadA-8e V106W) (Addgene plasmid 138495)^21^ were gifts from David Liu. The plasmid for pmCherry_gRNA was a gift from Ervin Welker (Addgene plasmid 80457). The N-terminal ABE8e and C-terminal ABE8e constructs were adapted from the Cbh_v5 AAV-ABE N terminus (Addgene plasmid 137177)^46^ and Cbh_v5 AAV-ABE C terminus (Addgene plasmid 137178)^46^ and synthesized by Twist Bioscience. The CK8e promoter,^47^ a gift from Stephen Hauschka, and the elongation factor 1α short (EFS) promoter, synthesized by Twist Bioscience, were cloned into AAV plasmids. Cloning was done using NEBuilder HiFi DNA Assembly (NEB) into restriction enzyme-digested destination vectors.

### Cell culture

HEK293T and C2C12 cells were maintained in Dulbecco’s modified Eagle’s medium (DMEM) supplemented with 10% (v/v) fetal bovine serum. For transfection experiments, cells were seeded onto 24 well plates at 125,000 cells per well. The following day, cells were transfected with plasmids using Lipofectamine 3000 according to the manufacturer’s instructions. Cells were harvested for downstream analyses 3 days later. For AAV transduction of C2C12 cells, cells were seeded onto 24 well plates at 125,000 cells per well. The following day, cells were infected with AAV at an MOI of 5 × 10^10^ vg/cell. C2C12 cells were differentiated into myotubes by replacing the medium with DMEM supplemented with 2% (v/v) horse serum.

### iPSC generation, maintenance, and differentiation

The *DMD* ΔEx48–50 iPSCs (RBRC-HPS0164) were purchased from Cell Bank RIKEN BioResource Center. The *DMD* ΔEx44 iPSCs and *DMD* ΔEx52 iPSCs were derived from two DMD patients by reprogramming peripheral blood mononuclear cells using Sendai virus at the UT Southwestern Wellstone Myoediting Core.^5^^,^^6^ iPSC culture and differentiation were performed as previously described.^40^ Briefly, iPSCs were cultured on Matrigel (Corning)-coated tissue culture polystyrene plates and maintained in mTeSR1 medium (STEMCELL) and passaged at 60%–80% confluency using Versene. iPSCs were differentiated into CMs at 60%–80% confluency by treatment with CHIR99021 (Selleckchem) in RPMI supplemented with ascorbic acid (50 μg/mL) and B27 without insulin (RPMI/B27−) for 24 h (from day 0 to day 1). At day 1, the medium was replaced with RPMI/B27−. At day 3, the cells were treated with RPMI/B27− supplemented with WNT-C59 (Selleckchem). At day 5, the medium was refreshed with RPMI/B27−. From day 7 onward, iPSC-CMs were maintained in RPMI supplemented with ascorbic acid (50 μg/mL) and B27 (RPMI/B27) with the medium refreshed every 3–4 days. Metabolic selection of CMs was performed for 6 days starting at day 10 by culturing cells in RPMI without glucose and supplemented with 5 mM sodium DL-lactate and CDM3 supplement (500 μg/mL *Oryza sativa*-derived recombinant human albumin, A0237; Sigma-Aldrich; and 213 μg/mL L-ascorbic acid 2-phosphate; Sigma-Aldrich). All CM studies were done at days >35.

### iPSC nucleofection

One hour before nucleofection, iPSCs were pretreated by adding 10 μM ROCK inhibitor, Y-27632 (Selleckchem), to the medium. iPSCs were then dissociated into single cells using Accutase (Innovative Cell Technologies). Approximately 800,000 iPSCs were resuspended in 82 μL of P3 Primary Cell Nucleofector Solution and 18 μL of Supplement 1 (P3 Primary Cell 4D-Nucleofector X Kit L; Lonza) and then mixed with 1.5 μg of pmCherry_gRNA plasmid containing the sgRNA and 4.5 μg of ABE plasmid. The mixture was then immediately loaded into a Nucleocuvette vessel (Lonza) and nucleofected on the 4D-Nucleofector X Unit (Lonza). After nucleofection, iPSCs were cultured in mTeSR Plus medium supplemented with 10 μM ROCK inhibitor and then switched to fresh mTeSR Plus medium the following day. Nucleofections were performed in triplicates, and a sample of iPSCs from each “pool” was evaluated for DNA editing efficiencies. For differentiation of edited iPSCs into CMs, the pools were combined into a single line.

### Immunocytochemistry

For iPSC-CMs, 1 × 10^5^ cells were seeded on 12 mm coverslips coated with poly-D-lysine and Matrigel (Corning) and fixed in cold acetone (10 min, −20°C). For C2C12 myotubes, cells were fixed on coverslips in 4% paraformaldehyde (PFA) (15 min, room temperature). Coverslips were blocked for 1 h with a blocking cocktail (2% normal horse serum/2% normal donkey serum/0.2% bovine serum albumin [BSA]/phosphate-buffered saline [PBS]). For iPSC-CMs, mouse anti-dystrophin (1:800) (MANDYS8; Sigma-Aldrich, D8168) and rabbit anti-troponin I (1:200) (clone H170; Santa Cruz Biotechnology; sc-15368) in 0.2% BSA/PBS were applied and incubated overnight at 4°C. For C2C12 myotubes, mouse anti-SpCas9 (1:800) (clone 7A9; Millipore Sigma; MAC133) and rabbit anti-laminin (1:200) (Sigma-Aldrich; L9393) were applied and incubated overnight at 4°C. The next day, cells were probed for 1 h with biotinylated horse anti-mouse immunoglobulin G (IgG) (1:200) (Vector Laboratories; BA-2000) and fluorescein-conjugated donkey anti-rabbit IgG (1:50) (Jackson ImmunoResearch; 711-095-152) diluted in 0.2% BSA/PBS. Unbound secondary antibodies were removed with PBS washes, and final dystrophin labeling was done with a 10 min incubation of rhodamine avidin DCS (1:60) (Vector Laboratories) diluted in PBS. Nuclei were labeled with DAPI (Sigma-Aldrich; D9542).

### Generation of adeno-associated viruses

AAVs were prepared by the Boston Children’s Hospital Viral Core. AAV vectors were purified by discontinuous iodixanol gradients (Cosmo Bio; AXS-1114542-5) and concentrated with a Millipore Amicon filter unit (UFC910008, 100 kDa). AAV titers were determined by quantitative real-time PCR assays.

### Mice

Mice were housed in a barrier facility with a 12 h:12 h light:dark cycle and maintained on standard chow (2916 Teklad Global). The ΔEx44 mouse model was generated previously in the C57BL/6J background.^5^ Briefly, mouse zygotes were microinjected with Cas9 mRNA and two sgRNAs targeting intronic regions around *Dmd* exon 44. Deletion was confirmed by DNA and cDNA Sanger sequencing and absence of dystrophin protein staining. For intramuscular injections, mice were first anesthetized by intraperitoneal injection of a ketamine and xylazine anesthetic cocktail. Intramuscular injection of P12 male ΔEx44 mice was performed via slow longitudinal injection into TA muscles using an ultrafine needle (31G) with 50 μL of saline solution or a prepared mixture of the dual AAV viruses (5 × 10^10^ vg of each virus per leg). For neonatal systemic injections, P2 male ΔEx44 mice were first lightly anesthetized on ice before injection into the superficial temporal facial vein^48^ using an ultrafine needle (31G) with 40 μL of a prepared mixture of the dual AAV viruses at indicated doses. For juvenile systemic injections, P21 male ΔEx44 mice were placed into a restrainer and injected into the tail vein using an ultrafine needle (31G) with 150 μL of a prepared mixture of the dual AAV viruses at indicated doses.

### Grip strength measurements

Grip strength of forelimb muscles was measured using a BIO-GS3 grip strength test meter (Bioseb Instruments). Mice were first weighed and then lifted by the tail to allow the forelimbs to grab a metal grid connected to the meter. The mouse was slowly pulled back in the horizontal plane until the grip was broken, and the force applied to the grid, just before loss of grip, was measured and recorded as force. Measurements were repeated five times for each mouse to determine average grip strength and were conducted by an experienced operator blinded to the experimental groups.

### Genomic DNA and RNA isolation and cDNA synthesis

Genomic DNA of iPSC-CMs, mouse skeletal muscles, and mouse hearts was isolated using a DNeasy Blood & Tissue Kit (Qiagen). RNA was isolated using the RNeasy Micro Kit (Qiagen). cDNA was reverse transcribed from RNA using iScript Reverse Transcription Supermix (Bio-Rad) according to the manufacturer’s protocol.

### On-target and off-target editing efficiency analysis

For Sanger sequencing, target sites were PCR amplified (Table S1) using PrimeStar GXL polymerase (Takara), and PCR cleanup was done using ExoSap-IT Express (Thermo Fisher). Chromatograms were analyzed using EditR to determine base editing efficiencies.^49^ Candidate OTSs were identified with CRISPOR, and the top five sites, by cutting frequency determination (CFD) score, for which PCR products were successfully obtained were selected.^50^^,^^51^ Target sites were PCR amplified (Table S1) using PrimeStar GXL polymerase (Takara), and a second round of PCR was used to add Illumina flow cell binding sequences and barcodes. PCR products were purified with AMPure XP beads (Beckman Coulter), analyzed for integrity on a 2200 TapeStation system (Agilent), and quantified by Qubit dsDNA high-sensitivity assay (Invitrogen) before pooling and loading onto an Illumina MiSeq. Following demultiplexing, resulting reads were analyzed with CRISPResso2 for editing frequency.^52^ To analyze the number of AAV integration events at the on-target site, we followed a previously established method.^53^ Sequencing files were aligned to AAV vector sequences using the bwa program (version 0.7.17) and sorted with samtools (version 1.6), and the number of reads that had vector sequences were counted and normalized to number of reads that mapped to the target amplicon.

### Western blot

iPSC-CMs or HEK293Ts were resuspended in lysis buffer (10% sodium dodecyl sulfate, 62.5 mM Tris [pH 6.8], 1 mM EDTA, and protease inhibitor). Mouse tissues were flash-frozen and crushed into a fine powder before being resuspended in lysis buffer. Protein concentration was determined by BCA assay, and 20–50 μg of total protein was loaded onto a 4%–20% acrylamide gel. Blots were then incubated with anti-dystrophin antibody (Sigma-Aldrich; D8168, 1:1000), anti-Cas9-N-terminal (Cell Signaling Technology; 7A9-3A3, 1:500), or anti-Cas9-C-terminal (Sigma Aldrich; 10C11-A12, 1:500) at 4°C overnight or with mouse anti-vinculin antibody (Sigma-Aldrich; V9131, 1:1,000) at room temperature for 1 h, followed by horseradish peroxidase antibody (Bio-Rad Laboratories) at room temperature for 1 h. Blots were developed using western blotting luminol reagent (Santa Cruz Biotechnology; sc-2048). Relative protein expression (densitometry) was measured using ImageJ’s Gel Analysis method, normalized to vinculin expression, and compared with the normalized WT dystrophin protein expression.

### Tissue histology

Tissues were individually dissected out. Skeletal muscles were cryoembedded in a 1:2 (vol:vol) mixture of gum tragacanth powder (Sigma-Aldrich) to tissue freezing medium (Triangle Bioscience). Heart muscles were cryoembedded in tissue freezing medium. All embeds were snap-frozen in isopentane heat extractant and supercooled to −155°C. A Leica CM3050 cryostat was used to prepare 8 μm transverse sections of muscles. H&E staining was performed according to established staining protocols. Dystrophin immunohistochemistry was performed using MANDYS8 monoclonal antibody (Sigma-Aldrich; D8168, 1:400). Image analyses were performed using Fiji software on at least three muscles for each condition as indicated. Myofiber diameter was calculated as minimal Feret’s diameter.

### Quantitative real-time PCR analysis and RT-PCR analysis

qRT-PCRs were assembled using Applied Biosystems TaqMan Fast Advanced Master Mix (Applied Biosystems). Primers (Table S1) were used to amplify the Δ44 *Dmd* transcript (includes exons 43–45–46) and the exon-skipped Δ44–45 *Dmd* transcript (includes exons 43–46–47) normalized to total *Dmd* transcript expression (includes exons 39–41). Assays were performed using Applied Biosystems QuantStudio 5 real-time PCR System (Applied Biosystems). For RT-PCR analysis, target exons were PCR amplified from cDNA using GoTaq polymerase (Promega) and amplicons were run on an agarose gel. To quantify splicing outcomes, PCR products were TOPO-TA cloned into a destination vector (Thermo Fisher Scientific). Minipreps (Qiagen) were performed on at least 20 colonies for each condition, and isolated plasmid was Sanger sequenced.

### Viral copy number assay

AAV viral copy number was determined by digital PCR of purified genomic DNA using custom-designed primers and TaqMan probes (Integrated DNA Technologies) (Table S1) on a QuantStudio Absolute Q digital PCR system (Thermo Fisher Scientific). The primers and probes anneal to the N-terminal and C-terminal Cas9 genes. A copy number reference assay for *Tfrc* (Thermo Fisher Scientific) was used to normalize genome copy number.

### Statistics

All data are presented as means ± SD. Ordinary one-way ANOVA was performed for comparison among the respective groups as indicated in the figures. Data analyses were performed with statistical software (GraphPad Prism software). p values less than 0.05 were considered statistically significant.

## Data availability

All data needed to evaluate the conclusions are present in the paper and supplementary material. DNA ADS files can be accessed at the National Center for Biotechnology Information Sequence Read Archive (NCBI SRA) with accession code PRJNA943277.

## Supplemental information

Document S1. Figures S1–S9

Table S1. Summary of oligos. Please see attached Excel file for sequences

Table S2. Sequences of AAV constructs. Please see attached Excel file for sequences

Document S2. Article plus supplemental information
