# Supplementary material for: Single-swap editing for the correction of common Duchenne muscular dystrophy mutations
Source: Mol Ther Nucleic Acids. 2023 Apr 19;32:522–35. doi: 10.1016/j.omtn.2023.04.009 (PMC10192335; doi:10.1016/j.omtn.2023.04.009)
Supplement: Document S1. Figures S1–S9 [file mmc1.pdf]

OMTN, Volume *32*

Supplemental information

Single-swap editing for the correction of common Duchenne muscular dystrophy mutations

Andreas C. Chai, Francesco Chemello, Hui Li, Takahiko Nishiyama, Kenian Chen, Yu Zhang, Efraín Sánchez-Ortiz, Adeeb Alomar, Lin Xu, Ning Liu, Rhonda Bassel-Duby, and Eric N. Olson

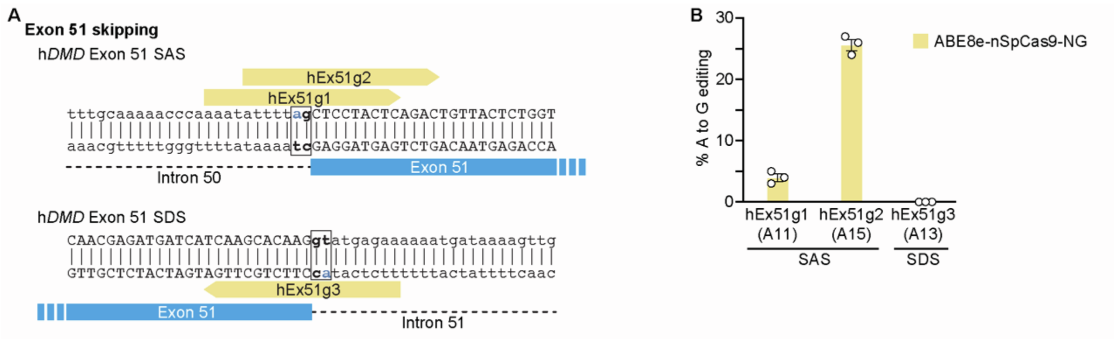

C

Target	Sequence	CFD score	Description	Locus
ON-T	ATTTTAGCTCCTACTCAGAC		SAS *DMD* exon 51	
OFF-T1	ATTTTAGATCCAACTCAGAC	0.52	intergenic_MIR492| NDUFA12	chr12_94948714
OFF-T2	TTTTCAGCTCATACTCAGTC	0.40	intergenic_KTN1| RP11-813I20.2	chr14_55703239
OFF-T3	CTTTTAGCTTCCACTCAGAA	0.38	intergenic_RN7SL61 8P|RP11-384G23.1	chr13_53271213
OFF-T4	ATTTGAGCACCTACTAAGTC	0.32	intron_C6orf163	chr6_87350917
OFF-T5	CTTTTAGCCCCTACTCACTC	0.20	intron_MAPK4	chr18_50655776

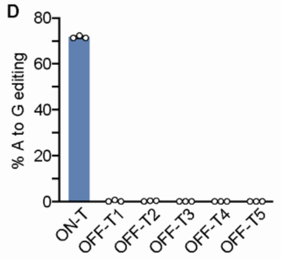

Figure S1. Identification of the most efficient 'single-swap' guide for exon skipping of *DMD* Exon 51. (A) Schematic of candidate sgRNAs for single-swap editing at the SAS or SDS of *DMD* exon 51. Target adenines in relation to candidate sgRNAs are indicated in blue. Canonical splice site motifs are in bold and boxed. **(B)** Editing efficiency by Sanger sequencing of target adenine using candidate sgRNAs with ABE8e-nSpCas9-NG following transient transfection in HEK293T cells. The editing efficiency of the most efficient guide, hEx51g2, is 25.7 ± 1.7% **(C)** Candidate off-target sites within the human genome for hEx51g2 as identified by CRISPOR and ranked by Cutting Frequency Determination (CFD) score. Target adenine of *DMD* exon 51 SAS is highlighted in blue. Corresponding adenines at off-target sites are highlighted in red. **(D)** Editing efficiency by Sanger sequencing of target adenines at on-target (71.6 ± 0.6%) and potential off-target sites (<0.2%) in ΔEx48-50 iPSCs. Data are mean ± s.d. *n* = 3 independent replicates.

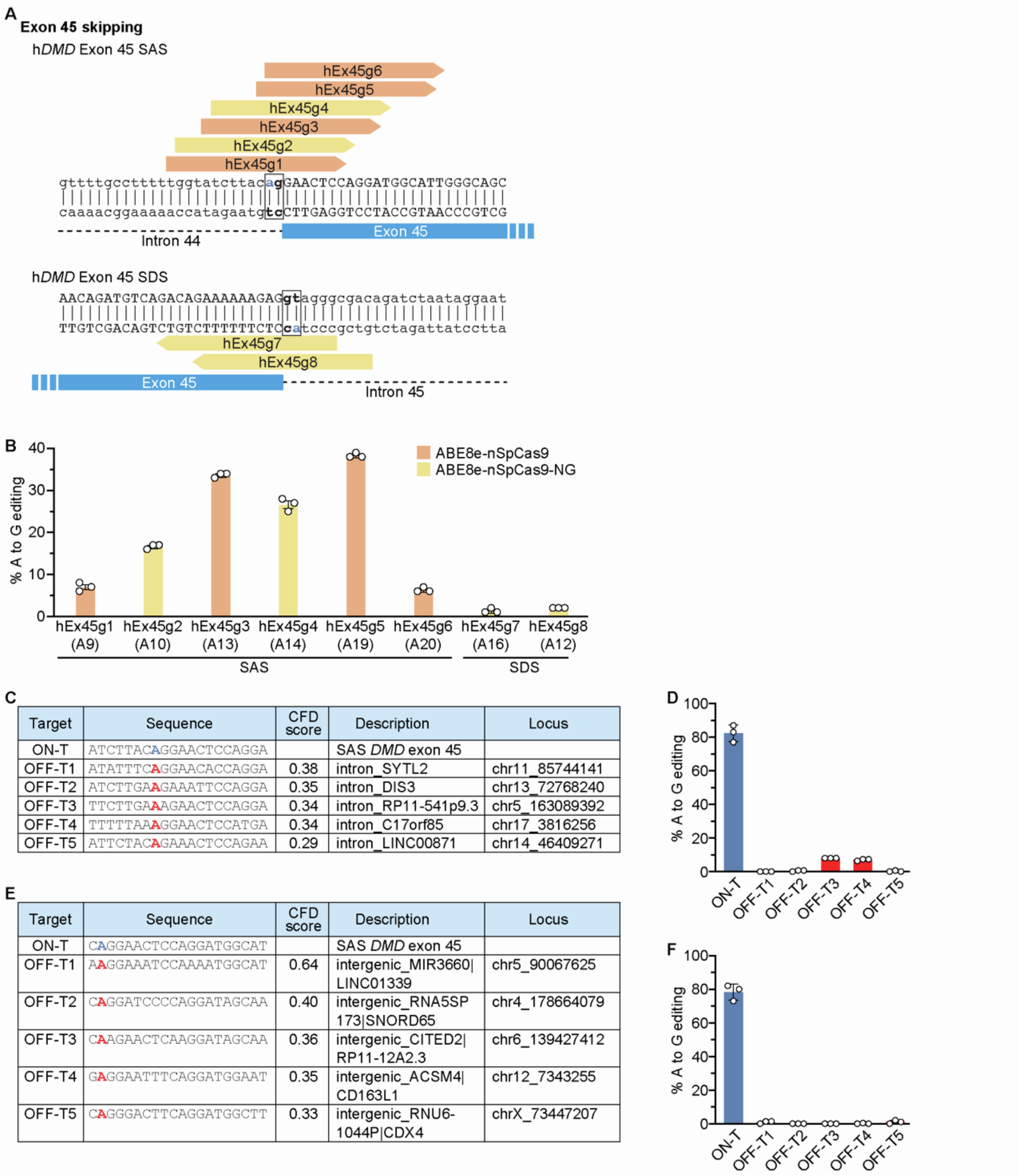

Target	Sequence	CFD score	Description	Locus
ON-T	ATCTTACAGGAACTCCAGGA		SAS *DMD* exon 45	
OFF-T1	ATATTTCAGGAACACCAGGA	0.38	intron_SYTL2	chr11_85744141
OFF-T2	ATCTTGAAGAAATTCCAGGA	0.35	intron_DIS3	chr13_72768240
OFF-T3	TTCTTGAAAGAACTCCAGGA	0.34	intron_RP11-541p9.3	chr5_163089392
OFF-T4	TTTTTAAAGGAACTCCATGA	0.34	intron_C17orf85	chr17_3816256
OFF-T5	ATTCTACAGAAACTCCAGAA	0.29	intron_LINC00871	chr14_46409271

Target	Sequence	CFD score	Description	Locus
ON-T	CAGGAACTCCAGGATGGCAT		SAS *DMD* exon 45	
OFF-T1	AAGGAAATCCAAAATGGCAT	0.64	intergenic_MIR3660|LINC01339	chr5_90067625
OFF-T2	CAGGATCCCCAGGATAGCAA	0.40	intergenic_RNA5SP173|SNORD65	chr4_178664079
OFF-T3	CAAGAACTCAAGGATAGCAA	0.36	intergenic_CITED2|RP11-12A2.3	chr6_139427412
OFF-T4	GAGGAATTTCAGGATGGAAT	0.35	intergenic_ACSM4|CD163L1	chr12_7343255
OFF-T5	CAGGGACTTCAGGATGGCTT	0.33	intergenic_RNU6-1044P|CDX4	chrX_73447207

Figure S2. Identification of the most efficient 'single-swap' guide for exon skipping of *DMD* exon 45. (A) Schematic of candidate sgRNAs for single-swap editing at the SAS or SDS of *DMD* exon 45. Target adenines in relation to candidate sgRNAs are indicated in blue. Canonical splice site motifs are in bold. **(B)** Editing efficiency by Sanger sequencing of target adenine using candidate sgRNAs with ABE8e-nSpCas9-NG or ABE8e-nSpCas9 following transient transfection in HEK293T cells. The editing efficiencies of the most efficient guides, hEx45g3

and hEx45g5, are 33.7 ± 0.6% and 38.3 ± 0.6%, respectively. **(C)** Candidate off-target sites within the human genome for hEx45g3 as identified by CRISPOR and ranked by CFD score. Target adenine of exon 45 SAS is highlighted in blue. Corresponding adenines at off-target sites are highlighted in red. **(D)** Editing efficiency of target adenines at on-target (83.3 ± 5.0%) and potential off-target sites for hEx45g3 in ΔEx44 iPSCs. Significant editing was found at OFF-T3 (8.0 ± 0.0%) and OFF-T4 (7.0 ± 0.6%) sites. n = 3 independent replicates. **(E)** Candidate off-target sites within the human genome for hEx45g5 as identified by CRISPOR and ranked by CFD score. Target adenine of exon 45 SAS is highlighted in blue. Corresponding adenines at off-target sites are highlighted in red. **(F)** Editing efficiency by Sanger sequencing of target adenines at on-target (79.3 ± 4.7%) and potential off-target sites (<1.0%) for hEx45g5 in ΔEx44 iPSCs. Data are mean ± s.d. n = 3 independent replicates.

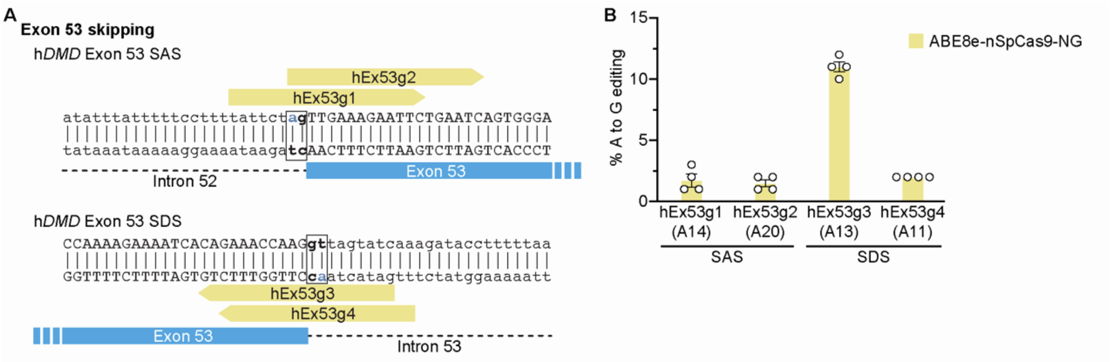

C

Target	Sequence	CFD score	Description	Locus
ON-T	GATACTAACCTTGGTTTCTG		SAS *DMD* exon 53	
OFF-T1	AATAATAATTTTGGTTTCTG	0.42	intergenic_RP11-328C8.5|PRICKLE1	chr12_42581943
OFF-T2	AATACTAACCTAGGTTACTA	0.36	intron_PDE4D	chr5_59387364
OFF-T3	GATAGAAAACATGGTTTCTG	0.33	intron_DNAJC16	chr1_15562847
OFF-T4	AATAATAACCTTAGTTTCTT	0.33	intergenic_Y_RNA|RP11-301G19.1	chr6_67625470
OFF-T5	TGTACTATCCTCGGTTTCTG	0.32	intergenic_NEDD9|RP11-716O23.1	chr6_11386505

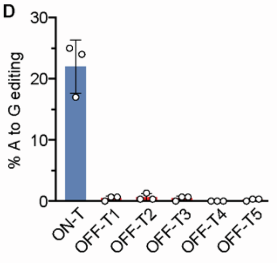

Figure S3. Identification of the most efficient 'single-swap' guide for exon skipping of *DMD* exon 53. (A) Schematic of candidate sgRNAs for single-swap editing at the SAS or SDS of *DMD* exon 53. Target adenines in relation to candidate sgRNAs are indicated in blue. Canonical splice site motifs are in bold. **(B)** Editing efficiency by Sanger sequencing of target adenine using candidates sgRNAs with ABE8e-nSpCas9-NG following transient transfection in HEK293T cells. The editing efficiency of the most efficient guide, hEx53g3, is 11.0 ± 0.8%. **(C)** Candidate off-target sites within the human genome for hEx53g3 as identified by CRISPOR and ranked by CFD score. Target adenine of exon 53 SDS is highlighted in blue. Corresponding adenines at off-target sites are highlighted in red. **(D)** Editing efficiency by Sanger sequencing of target adenines at on-target (22.0 ± 4.4%) and potential off-target sites (<0.7%) for hEx53g3 in ΔEx52 iPSCs. Data are mean ± s.d. *n* = 3 independent replicates.

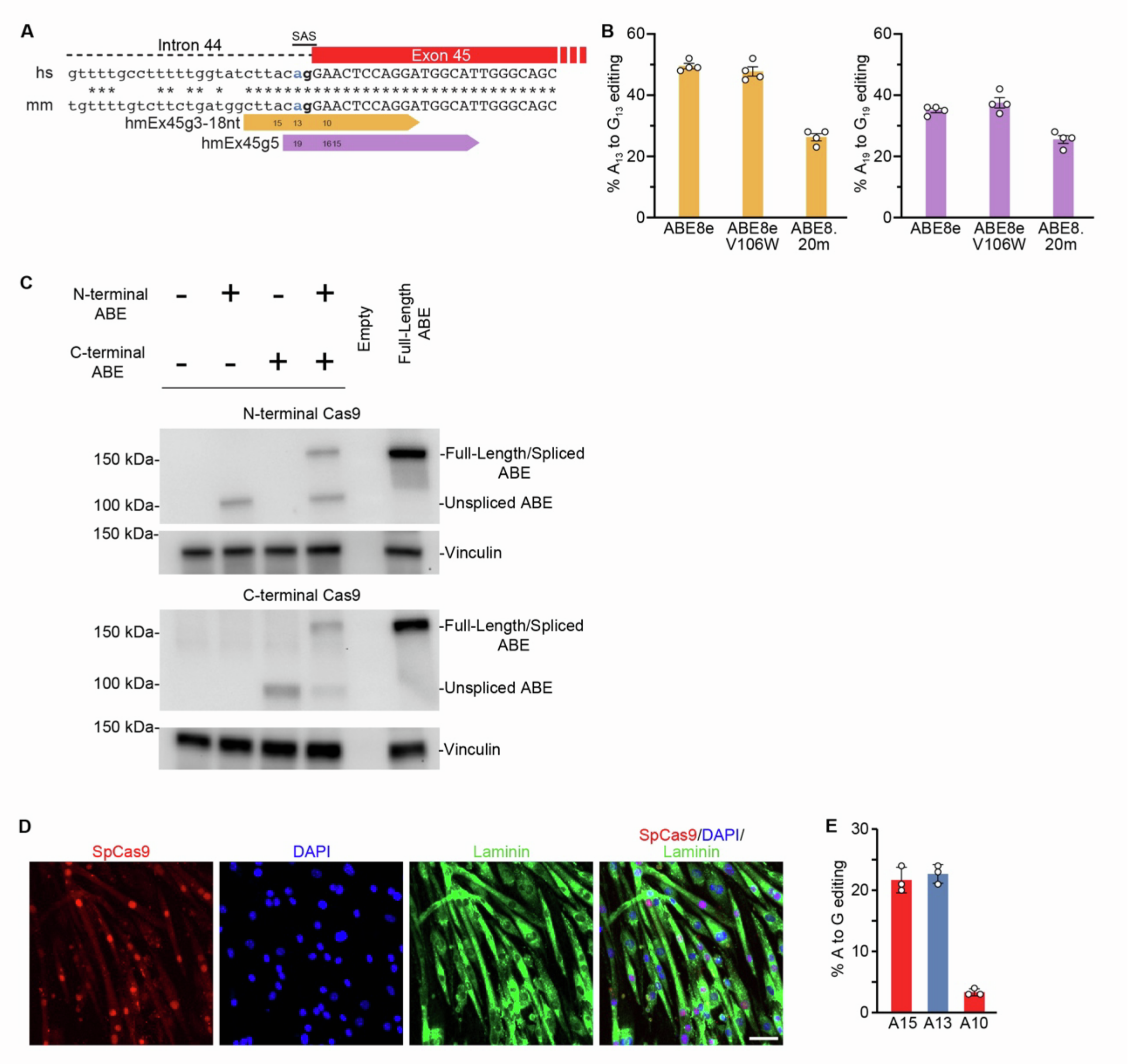

Figure S4. Identification of the most efficient 'single-swap' guide for exon skipping of *Dmd* exon 45. (A) Schematic of sgRNAs for single-swap editing at the SAS of *Dmd* exon 45 that can target both the human and mouse genome. Target adenines in relation to candidate sgRNAs are indicated in blue. Canonical splice site motifs are in bold. Homologous bases between the human (hs) and mouse (mm) genome are indicated by (*). **(B)** Editing efficiency by Sanger sequencing of target adenine using candidate engineered deaminases and sgRNA hmEx45g3-18nt (left: 26.3 ± 2.4% to 49.5 ± 1.7%) or sgRNA hmEx45g5 (right: 25.5 ± 2.5% to 37.5 ± 3.3%). **(C)** Western blots using antibodies for N-terminal Cas9 or C-terminal Cas9 showing ABE8eV106W-nSpCas9

expression and splicing in HEK293T cells transiently transfected with plasmids encoding the N-terminal ABE, the C-terminal ABE, or the full-length ABE. Vinculin is the loading control. Expected molecular weights: N-terminal ABE, 105 kDa; C-terminal ABE, 100 kDa; full-length ABE, 186 kDa; vinculin, 124 kDa. **(D)** Transduction of C2C12 myotubes with the dual AAV ABE8e system results in robust ABE8eV106W-nSpCas9 protein expression and localization to the nucleus as shown by immunocytochemistry. ABE8eV106W-nSpCas9 protein is stained in red (antibody against N-terminal half of SpCas9), DAPI stains nuclei in blue, and laminin outlines myotubes in green. Scale bar, 25 μm. **(E)** Editing efficiency by Sanger sequencing of editable adenines of genomic DNA from C2C12 myotubes infected with the dual AAV ABE8e system. Target adenine is colored in blue; editing efficiency is 22.7 ± 1.5%. Data are mean ± s.d. n = 3-4 independent replicates.

A

Target	Sequence	CFD score	Description	Locus
ON-T	**CTTACAGGAACTCCAGGA		SAS *Dmd* exon 45	
OFF-T1	AGCTTACGGGAACTCAAGGT	0.39	exon_Plxna3	chrX_73375138
OFF-T2	GACTCACCGGAACTCCAGGA	0.36	exon_Col7a1	chr9_108799469
OFF-T3	GGCCCAAAGGAACTCCAGAA	0.34	intron_Col4a2	chr8_11436024
OFF-T4	CACTTATAGGAATTCCAGGA	0.34	exon_Lgals9	chr11_78854104
OFF-T5	TTCTCACAGAAACTCCAGGA	0.15	intron_Ikzf3	chr11_98406640

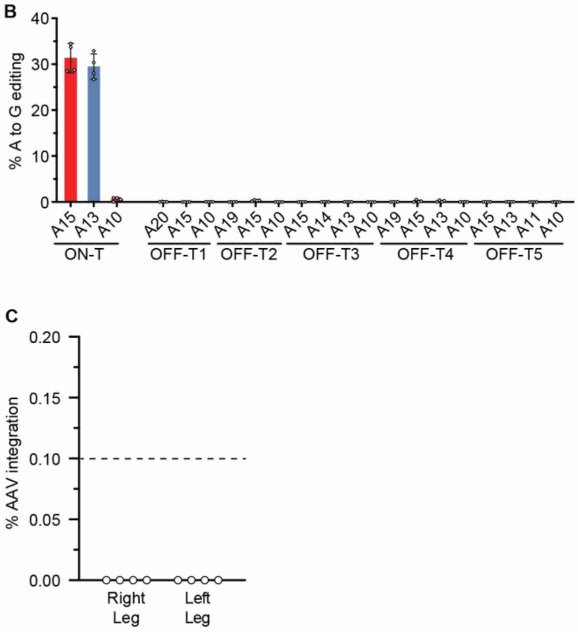

Figure S5. Off-target editing for hmEx45g3-18nt in the mouse genome. (A) Candidate off-target sites within the mouse genome for hmEx45g3-18nt as identified by CRISPOR. Target adenine of exon 45 SAS is highlighted in blue. Potential bystander adenines are highlighted in red. Editable adenines at off-target sites are highlighted in bold. **(B)** Editing efficiency by amplicon deep sequencing of editable adenines at on-target and potential off-target sites from the TA of mice treated with dual AAV ABE8e by IM injection. Target adenine is colored in blue; editing efficiency is 29.5 ± 2.7%. Off-target editing is <0.3% for all sites. **(C)** AAV integration frequency (<0.1%) at the on-target site from the TA of saline-treated right legs and dual AAV ABE8e-treated left legs of ΔEx44 mice. Data are mean ± s.d. n = 4 mice.

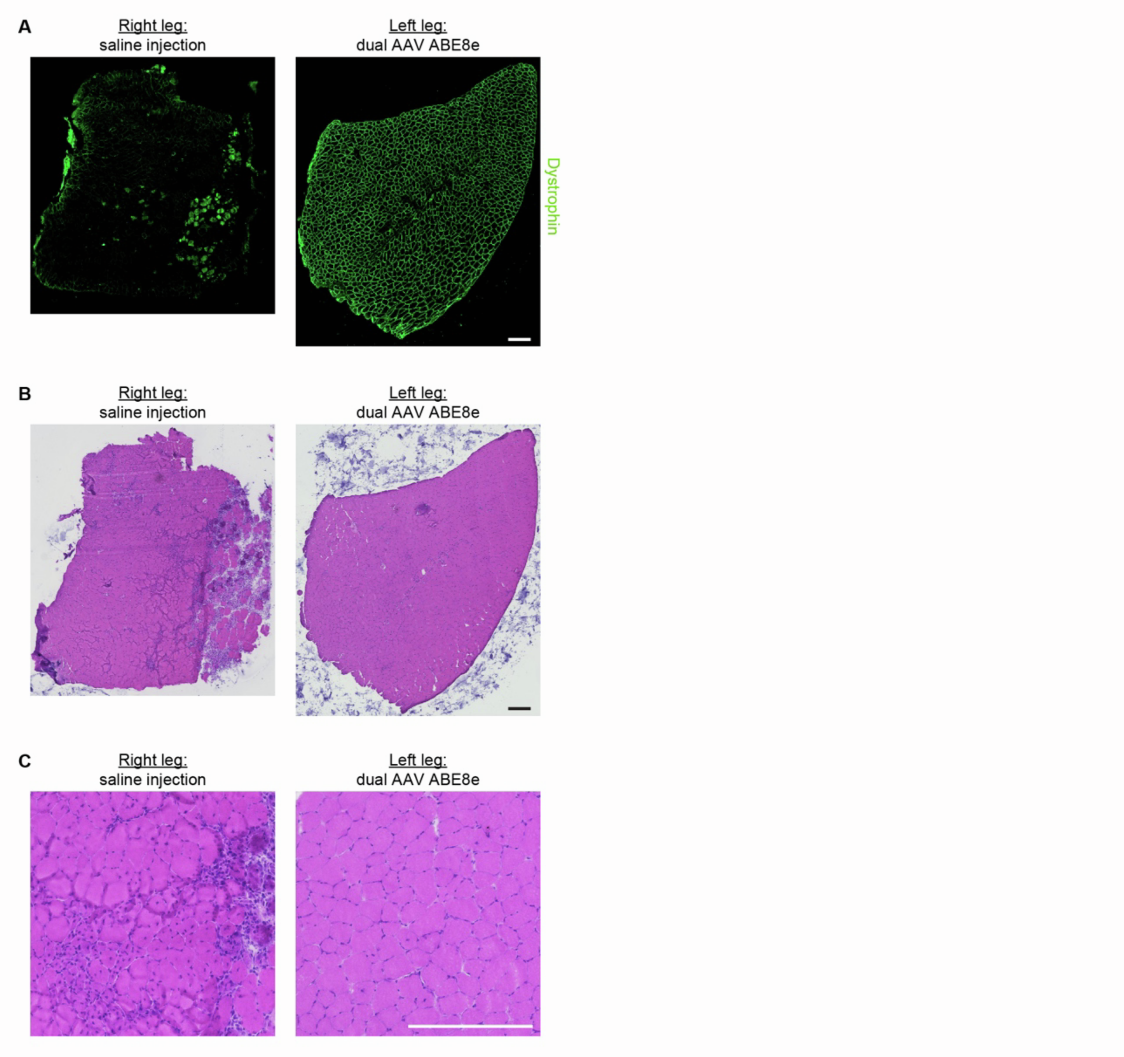

Figure S6. Representative immunohistochemistry and histological staining from a ΔEx44 mouse treated with saline and dual AAV ABE8e. (A) Immunohistochemistry for dystrophin of TA muscles from the saline-treated right leg and dual AAV ABE8e-treated left leg of a ΔEx44 mouse. Scale bar, 100 µm. Dystrophin is stained in green. **(B)** H&E staining of TA muscles from the saline-treated right leg and dual AAV ABE8e-treated left leg of a ΔEx44 mouse at 4x magnification and **(C)** 20x magnification. Scale bar, 100 µm.

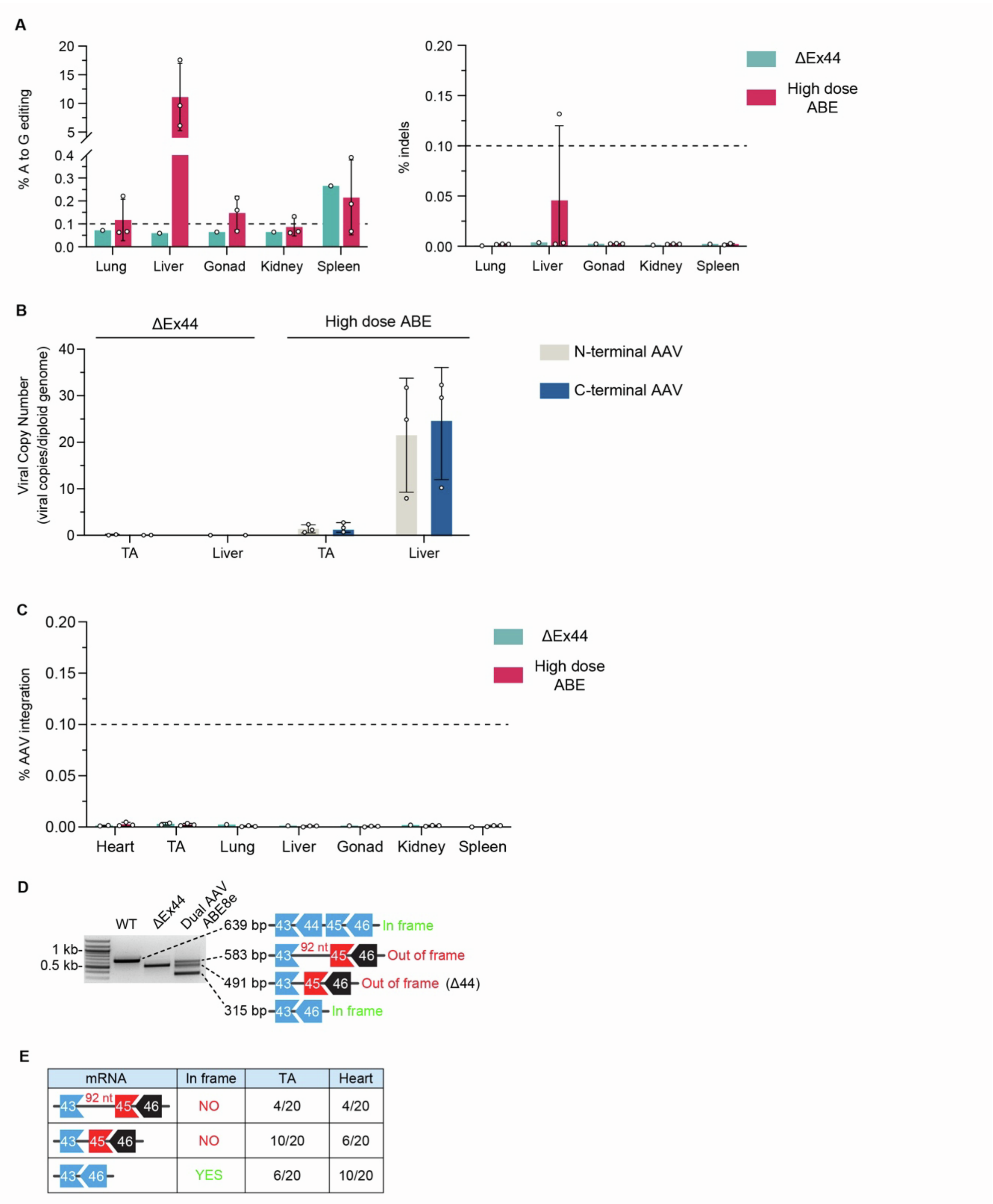

Figure S7. Editing analysis in ΔEx44 mice treated by systemic injection of dual AAV ABE8e. (A) Amplicon deep sequencing of DNA editing and indel frequency in lung, liver, gonad, kidney, and spleen tissue from high dose dual AAV ABE8e-treated and saline-treated ΔEx44 mice. Editing in the liver is 11.1 ± 5.9%, and <0.2% for all other tissues. Indel frequency

is <0.1% in all tissues. **(B)** Viral copy number of the N-terminal and C-terminal AAV halves from the liver (21.5 ± 12.3 N-terminal; 24.0 ± 12.0 C-terminal) and TA (1.4 ± 0.9 N-terminal; 1.7 ± 1.0 C-terminal) of high dose dual AAV ABE8e-treated and saline-treated Δex44 mice, normalized to the diploid genome. **(C)** AAV integration frequency (<0.1%) at the on-target site in heart, TA, lung, liver, gonad, kidney, and spleen tissue of high dose dual AAV ABE8e-treated and saline-treated ΔEx44 mice. **(D)** RT-PCR analysis of mRNA from the heart of a WT mouse, a ΔEx44 mouse, and a ΔEx44 mouse treated with dual AAV ABE8e run. The cDNA of the WT is 639 bp; of the ΔEx44 is 491 bp; of the ΔEx44 with the intronic inclusion is 583 bp; of the ΔEx44 with exon skipping of exon 45 is 315 bp. **(E)** Frequency of transcript splicing events from the TA and heart of a ΔEx44 mouse treated with dual AAV ABE8e by TOPO-TA analysis. *n*=1-3 mice.

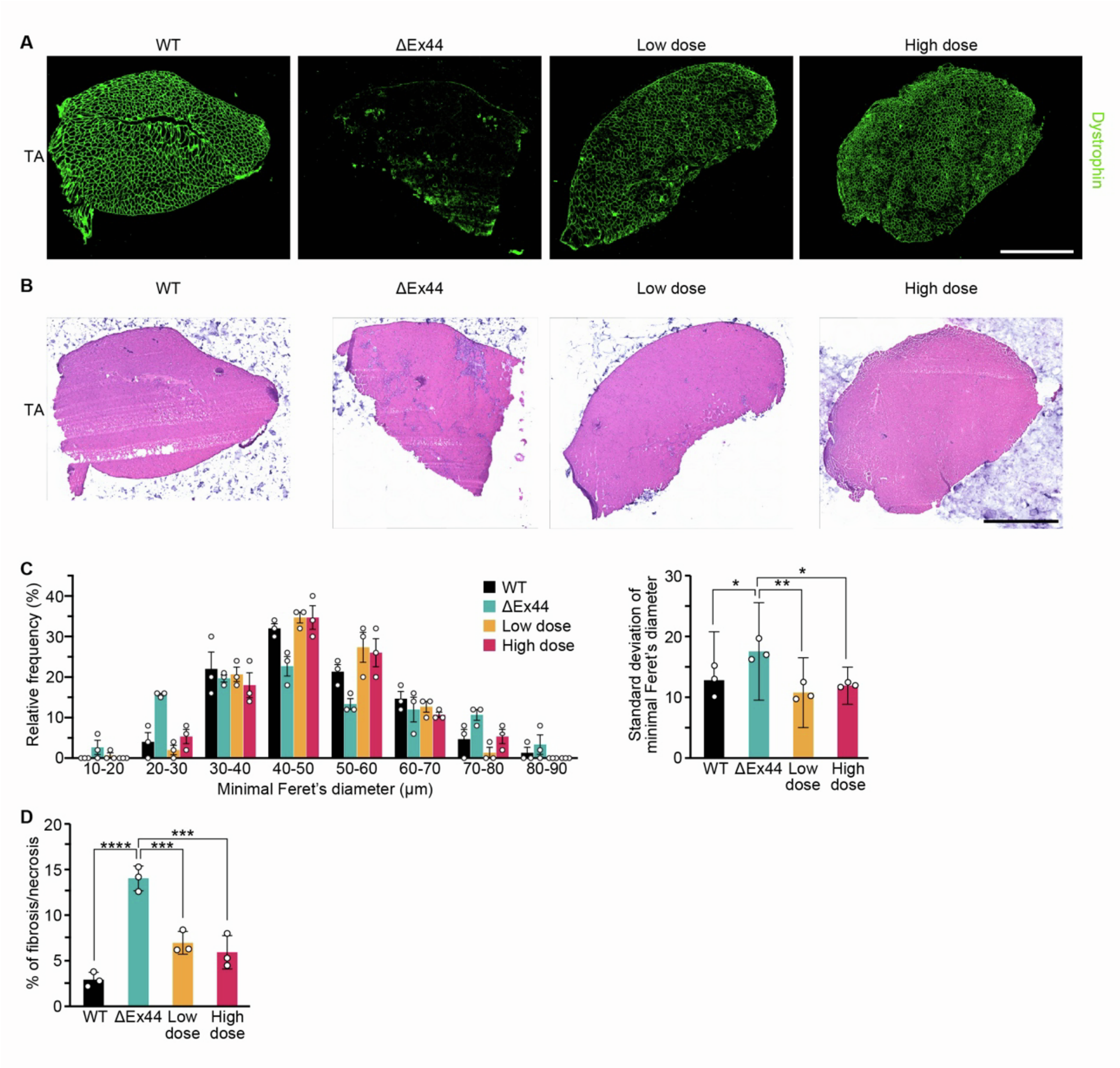

Figure S8. Representative immunohistochemistry and histological staining from ΔEx44 mice treated with dual AAV ABE8e. (A) Immunohistochemistry for dystrophin and **(B)** H&E staining of the TA of a WT mouse, a ΔEx44 mouse, and a ΔEx44 mouse treated with dual AAV ABE8e at the low and high doses. Scale bar, 1000 µm. Dystrophin is stained in green. **(C)** Histogram of minimal Feret's diameter of myofibers and standard deviations, (Data are mean ± s.e.m.) (WT, 12.8 ± 8.0 µm; ΔEx44, 17.5 ± 8 µm; ΔEx44 low dose, 10.8 ± 5.7 µm; ΔEx44 high dose, 11.9 ± 3.0 µm) and **(D)** percent area fibrosis or necrosis of TA muscles of a WT mouse (2.9 ± 0.8%), a ΔEx44 mouse (14.0 ± 1.4%), and a ΔEx44 mouse treated with dual AAV ABE8e at

the low (7.0 ± 1.2%) and high (5.9 ± 1.8%) doses. $n = 3$ mice. Data are mean ± s.d. *$p<0.05$, **$p<0.01$, ***$p<0.001$, ****$p<0.0001$ by ordinary one-way ANOVA.

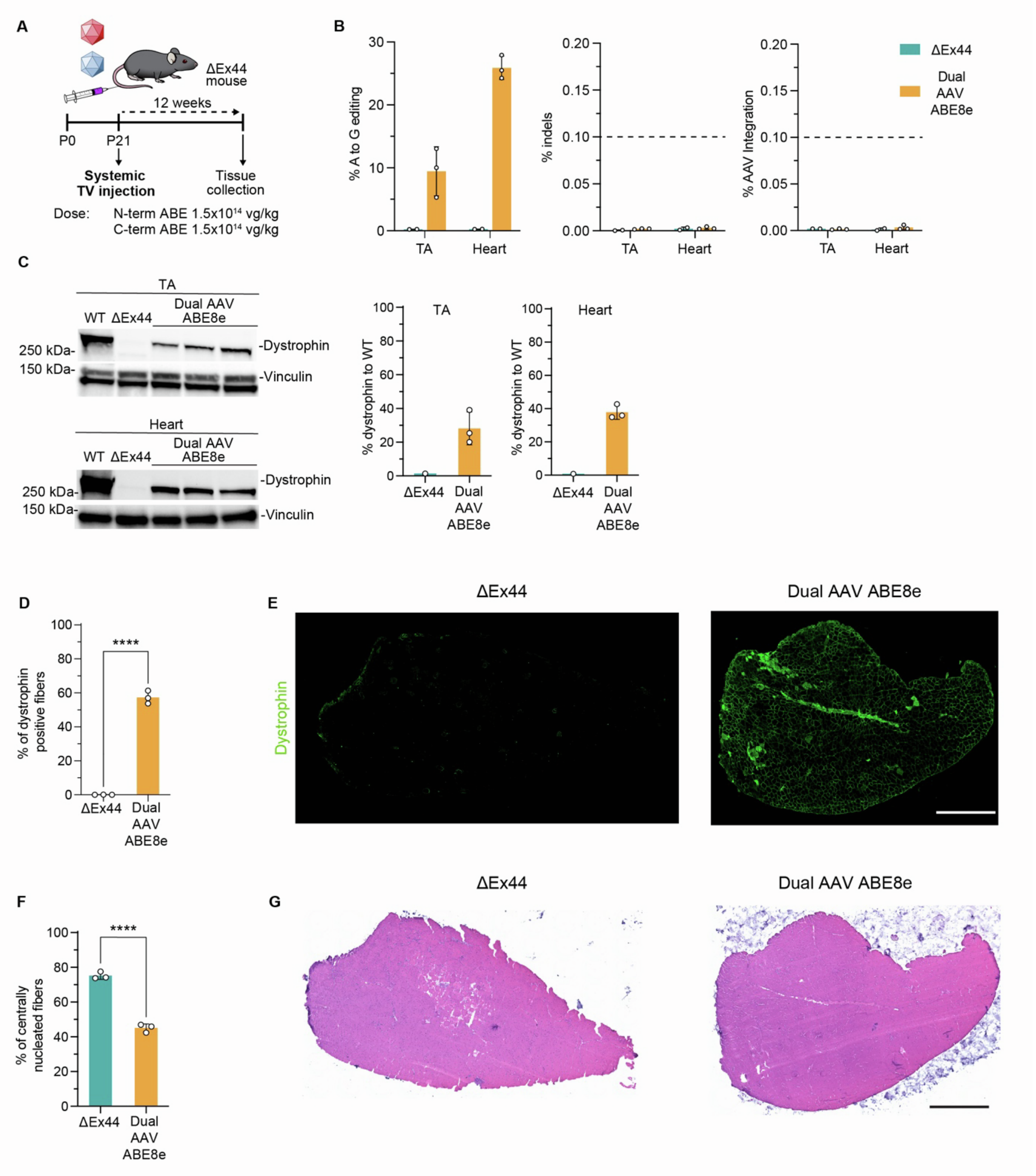

Figure S9. Treatment of juvenile P21 ΔEx44 mice with dual AAV ABE8e. (A) DNA editing efficiency in the heart (29.0 ± 1.7%) and TA (10.3 ± 2.5%), indel frequency (<0.1%), and AAV integration frequency (<0.1%) by amplicon deep sequencing and **(B)** dystrophin protein expression in the heart (28.2 ± 9.8%) and TA (37.8 ± 4.3%) of P21 ΔEx44 mice treated with dual

AAV ABE8e**.** Vinculin is the loading control. Relative intensity is measured as dystrophin expression normalized to vinculin compared to the WT. Western blots are quantified on the right panel. **(C)** The percentage of dystrophin positive fibers quantified from **(D)** immunohistochemistry sections of TAs from untreated (0.1 ± 0.1%) and dual AAV ABE8e treated ΔEx44 mice (57.3 ± 3.8%). Dystrophin is stained in green. **(E)** The percentage of centrally nucleated fibers from **(F)** H&E sections of TAs from untreated (75.2 ± 2.1%) and dual AAV ABE8e treated ΔEx44 mice (45.1 ± 2.4%). Scale bar, 1000 µm. Data are mean ± s.d. n = 1-3 mice. ****p<0.0001 by unpaired t-test.

Table S1. Summary of oligos. Please see attached Excel file for sequences.

Table S2. Sequences of AAV constructs. Please see attached Excel file for sequences.
